# Humid and cold forest connections in South America between the eastern Andes and the southern Atlantic coast during the LGM

**DOI:** 10.1038/s41598-024-51763-8

**Published:** 2024-01-24

**Authors:** Jorge Luiz Diaz Pinaya, Nigel C. A. Pitman, Francisco William Cruz, Thomas K. Akabane, Maria del Carmen Sanz Lopez, Augusto José Pereira-Filho, Carlos H. Grohman, Luiza Santos Reis, Erika S. Ferreira Rodrigues, Gregório C. T. Ceccantini, Paulo Eduardo De Oliveira

**Affiliations:** 1 Institute of Geosciences, University of São Paulo, São Paulo, Brazil; 2 Polytechnic School, University of São Paulo, São Paulo, Brazil; 3 Science Action, The Field Museum of Natural History, Chicago, IL USA; 4 Instituto de Astronomia, Geofísica e Ciências Atmosféricas, Universidade de São Paulo, São Paulo, Brazil; 5 Institute of Energy and Environment, University of São Paulo, São Paulo, Brazil; 6 Department of Botany, Institute of Biosciences, University of São Paulo, São Paulo, Brazil

**Keywords:** Biodiversity, Biogeography, Climate-change ecology, Ecological modelling, Forest ecology, Palaeoecology, Biogeography, Climate-change ecology, Ecological modelling, Forest ecology, Palaeoecology, Computational models, Statistical methods, Climate change, Palaeoclimate, Computational biology and bioinformatics, Ecology, Climate sciences, Ecology

## Abstract

The presence of Andean plant genera in moist forests of the Brazilian Atlantic Coast has been historically hypothesized as the result of cross-continental migrations starting at the eastern Andean flanks. Here we test hypotheses of former connections between the Atlantic and Andean forests by examining distribution patterns of selected cool and moist-adapted plant arboreal taxa present in 54 South American pollen records of the Last Glacial Maximum (LGM), ca. 19–23 cal ka, known to occur in both plant domains. Pollen taxa studied include *Araucaria*, *Drimys*, *Hedyosmum*, *Ilex*, *Myrsine*, *Podocarpus*, *Symplocos*, *Weinmannia*, Myrtaceae, Ericaceae and Arecaceae. Past connectivity patterns between these two neotropical regions as well as individual ecological niches during the LGM were explored by cluster analysis of fossil assemblages and modern plant distributions. Additionally, we examined the ecological niche of 137 plant species with shared distributions between the Andes and coastal Brazil. Our results revealed five complex connectivity patterns for South American vegetation linking Andean, Amazonian and Atlantic Forests and one disjunction distribution in southern Chile. This study also provides a better understanding of vegetation cover on the large and shallow South American continental shelf that was exposed due to a global sea level drop.

## Introduction

A long-standing biogeographic problem in the study of Brazilian vegetation is the occurrence of several Andean genera in the Montane Atlantic Rainforest of southern Brazil^1,2^. The presence of *Berberis* L.*, Clethra* Gronov. Ex L., *Crinodendron* Molina*, Daphnopsis* Mart., *Drimys* J.R. Forst. & G. Forst., *Escallonia* Mutis ex L.f., *Griselinia* Foster & Foster*, Gunnera* L., *Podocarpus* L’Hér. ex Pers., *Weinmannia* L., and Ericaceae in Brazil’s Atlantic Rainforest has been interpreted since the early 1950’s as an impoverished subset of the Andean flora which migrated eastwards during former cooler climates^1,2^. This hypothesis is supported by pollen records of glacial age^3–7,63^ indicating that during the last glacial cycles cold-adapted forests expanded westwards into the Brazilian cerrado, especially under cooler temperatures during the Last Glacial Maximum (LGM), ca. 19–23 cal ka, a time period interpreted as cold and humid in the Central Andes^8–11^ with high water levels in Lake Titicaca, whereas in Eastern Bolivian Cordillera a dry LGM^12^.

To further investigate the possible role of other areas of contact between these two floras during cold phases of the last glacial cycle we modeled past distribution patterns of selected cool and moist-adapted arboreal taxa during the LGM. This period of the last glacial cycle, characterized by ice sheet expansions in the northern hemisphere and in Antarctica^13–15^, is now interpreted as a phase of relatively high humidity and relatively lower temperatures in southern and southeastern Brazil^16–19^, simultaneously with humid and drier phases in southern and northern Chile, respectively^20,21^.

We hypothesize that the modern presence of Andean plant taxa in montane areas, i.e. with elevations > 600 m, of the Brazilian Atlantic Rainforest domain is the combined result of former connectivities supported by various climatic and biogeographical events during the LGM.

We propose that these connectivity patterns were established under past climate scenarios with relatively high levels of humidity associated with significant temperature depression and an atmosphere with relatively low levels of CO_2_^22^. The magnitude of this cooling phase was previously established to be in the order of 5° to 6 °C for the equatorial tropics^15,23–25^ and in the lowlands^26^, 4 °C to 8 °C in the southern Andes of Chile^15,27^ and in northern Argentina^28^. However, reanalyses of global temperature during the LGM appoint to -4 to -3 °C temperature depression in tropical low latitudes^29,30^. Important biogeographical events during this glacial phase also include downslope expansion of cold-adapted Andean plant taxa into the neotropical lowlands^31–33^, migration of montane rainforest taxa into Central and Southern Brazil^5^, and the exposure of the Atlantic Continental Shelf caused by a global sea-level drop of ca. 120–150 m^34–38^.

Inferring vegetation responses during the LGM involves understanding the species' niche concept, which encompasses complex abiotic and biotic variables which are beyond the goals of this research which focuses mainly on past distribution of selected indicator taxa under the light of humidity and temperature data that can be obtained directly from their ecology. Consequently, our study does not investigate the important role of atmospheric CO_2_ concentration and other significant paleoclimatic forcings in former plant distributions.

## Methods

Our methodological strategy to reconstruct potential phytogeographic connections in South America during the LGM involved: selection of pollen taxa and fossil records, ecological niche determination by means of potential distribution modelling (MaxEnt) during the LGM and their modern geographical occurrences to strengthen observed patterns, cluster analyses of LGM pollen records taking into account only selected taxa, modern ecological niche analysis of 137 Andean taxa with species occurring in the Atlantic domain to identify present-day relict connectivity populations, cluster analyses of present-day distributions of six key arboreal species found in both ecosystems to observe the presence of relicts in modern landscapes and their relative climatic ranges. Each methodological step is given in detail as follows:

The arboreal taxa selected for our pollen analysis are frequently found in glacial pollen spectra of tropical America, in most cases with abundance higher than 5%, and represent genera and families adapted to cold/mild and cold-humid conditions. They are: *Araucaria* Juss., *Drimys* J.R. Forst. & G. Forst., *Hedyosmum* Sw., *Ilex* L., *Myrsine* L., *Podocarpus* L’Hér. ex Pers., *Symplocos* Jacq., *Weinmannia* L., Myrtaceae Juss., Ericaceae Juss. and Arecaceae Bercht. & J. Presl; (*syn.* Palmae Juss., palms). Another advantage of these taxa is that they represent arboreal genera with high potential of long-distance migration via anemophilous (airborne) pollination and seed dispersal by birds and other animals^5^, which also imply increased ecological amplitude and greater colonization ability. Moreover, most of these genera are found along a range of plant successional gradients^5^.

Although ericaceous and palm pollen can be assigned to different genera, they are frequently represented in pollen diagrams simply as Ericaceae and Arecaceae. Pollen of Myrtaceae, a stenopalynous taxon, represents a large family with thousands of species whose triangular-shaped pollen cannot be distinguished at the species level under light microscopy. Cold- and humid-adapted species of Myrtaceae, Ericaceae and Arecaceae were verified in herbarium collections of the Field Museum of Natural History (F), of the Department of Botany of the Institute of Biological Sciences of the University of Sao Paulo (SPF) and remotely in the Flora do Brasil^39^. A total of 17 genera and 53 species within Myrtaceae, and 5 genera and 19 species in Arecaceae appear restricted to high elevations or occur at high latitudes in Brazil^5^ whereas Ericaceae is, in the tropics, an almost exclusively montane family^5^. To reduce the overrepresentation of myrtaceous taxa, our depiction of its modern distribution during the LGM is based on well-known cold/humid-adapted species such as *Siphoneugena densiflora* O.Berg, *Myrciaria floribunda* (H.West ex Willd.) O.Berg, *Myrciaria tenella* (DC.) O.Berg, *Pimenta pseudocaryophyllus* (Gomes) Landrum together with *Campomanesia eugenioides* (Cambess.) D.Legrand ex Landrum*, Myrcia loranthifolia* (DC.) G.P.Burton & E.Lucas (sin. *Calyptranthes grandifolia* O.Berg) and *Blepharocalyx salicifolius* (Kunth) O.Berg.

**Figure 1:**
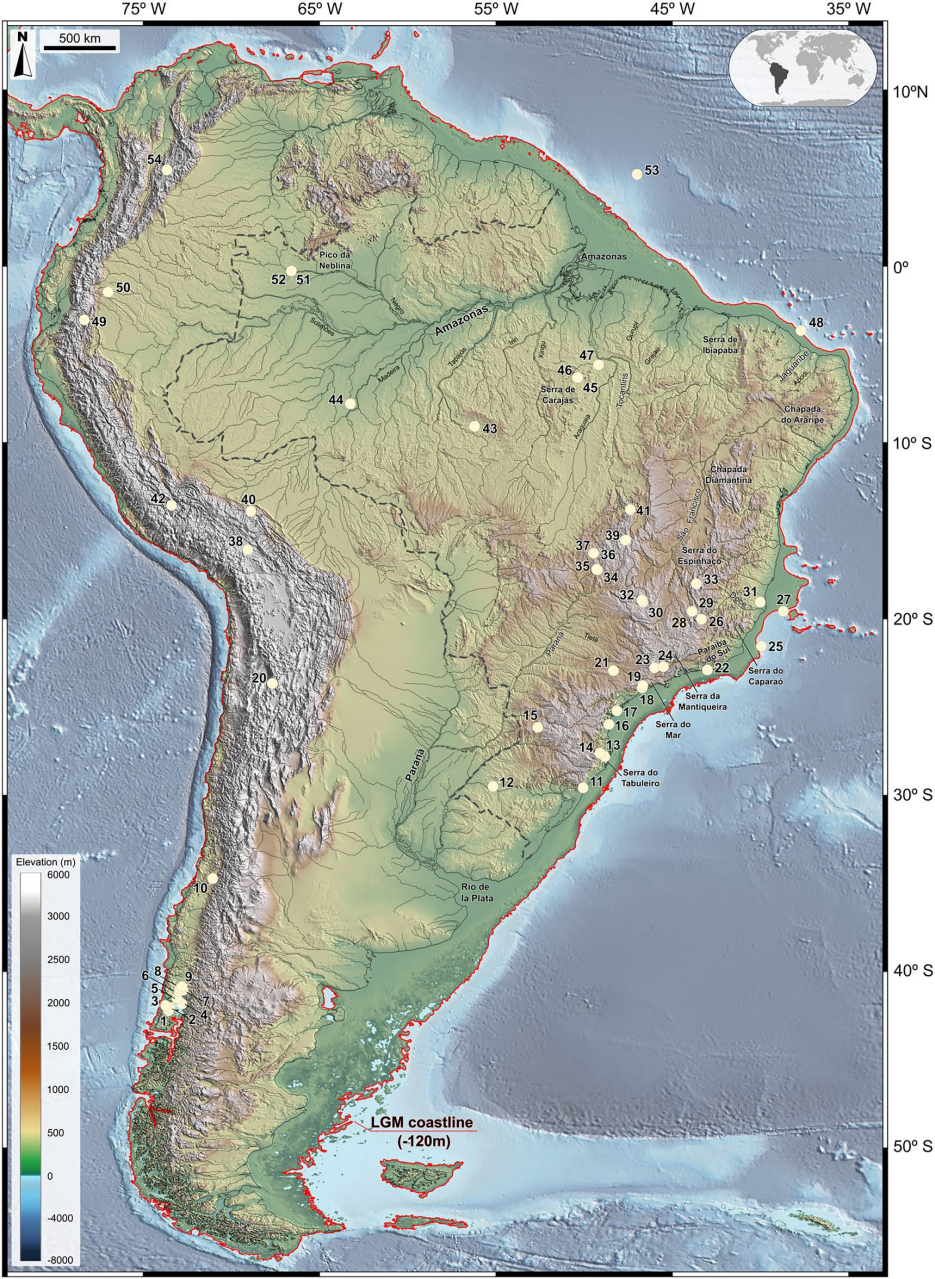
Map of South America during the LGM, showing 54 pollen record sites used in this study (white dots). The ancient shoreline is delimited by a red line, based on sea level ca. 120 m lower than present. Note the Brazilian Highlands with elevations ranging from ca. 600 to 2890 m connecting. The base maps are shaded relief images of the ETOPO1 Global Digital Elevation Model with 01-min spatial resolution, with custom hypsometric color scales. Raster shading and color scale creation were performed in GRASS-GIS 8.3 (https://grass.osgeo.org), map composition in QGIS 3.28 (https://qgis.org) and final artwork in Inkscape 1.3 (https://inkscape.org).

Vegetational and climatic reconstructions for the LGM were inferred from 50 continental pollen records obtained in exclusively closed sedimentary basins—to avoid uncertainties regarding pollen provenance—from Brazil, Bolivia, Chile, Colombia, Ecuador, Peru and Venezuela and 4 marine records from the South Atlantic (Fig. 1).

The list of selected sites are as follows. Continental records: 1. Dalcahue^27^; 2. Taiquemó (core HE94-2B), Isla Grand de Chiloe, Chile^40^; 3. Rio Negro profile, Isla de Chiloé, Chile^41^; 4. Canal de la Puntilla (core PM13), Chile^42^; 5. La Campana^27^; 6. Fundo Llanquihue^14^; 7. Alerce III, Chile^43^; 8. Fundo Lina Pantanosa, Chile^27^; 9. Canal de la Puntilla ou Puerto Octay spillway, Chile^44^; 10. Laguna Tagua-Tagua^20^; 11. Cambará do Sul^45^; 12. São Francisco de Assis^46^; 13. Serra do Tabuleiro^47^; 14. Serra da Boa Vista^48^; 15. Pato Branco^49^; 16. Volta Velha^50^; 17. Ilha do Cardoso^51,52^; 18. Curucutu^53^; 19. Colônia crater—Serra do Mar^54^; 20. Laguna Miscanti, Chile^21^; 21. Serra de Botucatu^55^; 22. Lagoa de Itaipu^56^; 23. Monte Verde^57^; 24. Morro de Itapeva^58^; 26. Catas Altas^59^; 28 and 29. Lagoa dos Olhos^6,7,16,18,139^; 30. Serra do Salitre^4^; 31. Brejo do Louro^60,61^; 32. Serra Negra^3,16^; 33. Serra do Espinhaço^62,63^; 34 and 35. Crominia^64,65^; 36 and 37. Turfa de Inhumas^66,67^; 38. Lake Titicaca, Peru and Bolivia^68^; 39. Lagoa Bonita^69^; 40. Lake Consuelo, Peru^70^; 41. Chapada dos Veadeiros^71^; 42. Lake Pacucha, Peruvian Andes^72^; 43. Lago do Saci^73^; 44. Humaitá^74^; 45. Serra Sul dos Carajás^75,76^; 46 and 47. Serra dos Carajás^75,76^; 49. San Juan Bosco^31^; 50. Mera^32^; 51 and 52. Lagoa da Pata^24,77^; 54. Lake Fúquene (Core Fúquene-7), Colombia^78,79^; Marine pollen records: 25. GeoB 3202–1^80^; 27. GeoB 3229-2^80^; 48. Jaguaribe River Delta—GeoB 3104–1^81^; 53. Amazon River Delta—ODP Hole 932A^82^.

Chronological calibration of pollen records was carried out with SHCAL13 Southern Hemisphere Calibration^83^, CALIB Radiocarbon Calibration version 7.1^84^ (http://calib.qub.ac.uk/calib/). The geographical distribution of all pollen records is a shaded relief image of the ETOPO1 Global Digital Elevation Model^85^ with 1-min spatial resolution, draped by a custom hypsometric color scale. For the continental area, shaded relief illumination is from 060°N, 30° above the horizon, with 40 times vertical exaggeration. In the oceanic area, illumination is from 060°N, 20° above the horizon, with 5 times vertical exaggeration. Raster shading and color scale creation^86^ were performed in GRASS-GIS 8.3 (https://grass.osgeo.org), map composition in QGIS 3.28 (https://qgis.org) and final artwork in Inkscape 1.3 (https://inkscape.org).

**Figure 2:**
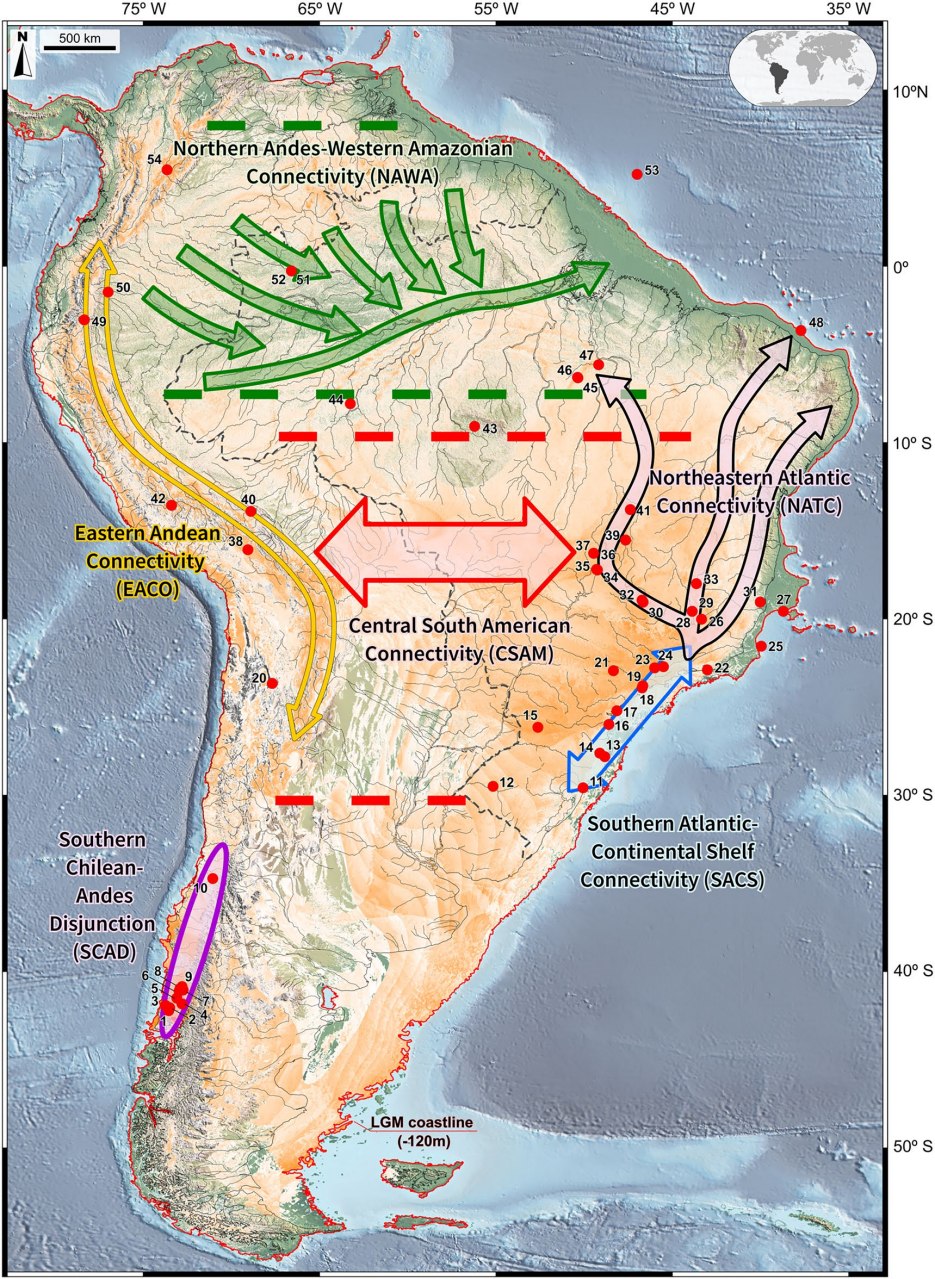
Map of South America during the LGM displaying five connectivity patterns and one biogeographical disjunction of vegetation: Central South American Connectivity (CSAM), Northern Andes-Western Amazonian Connectivity (NAWA), Southern Atlantic Continental Shelf Connectivity (SACS), Eastern Andean Connectivity (EACO), Northeastern Atlantic Connectivity (NATC) and Southern Chilean Andes Disjunction (SCAD). Also shown are the overlapping potential LGM distributions of *Araucaria*, Arecaceae, *Drimys*, Ericaceae, *Hedyosmum*, *Ilex*, *Myrsine*, Myrtaceae, *Podocarpus*, *Symplocos* and *Weinmannia* (orange to white *i.e.* high to low suitability). In addition shown 54 pollen record sites (red dots). The LGM coastline is delimited by a red line**,** based on sea level ca. 120 m lower than present. The base maps are shaded relief images of the ETOPO1 Global Digital Elevation Model with 01-min spatial resolution, with custom hypsometric color scales. Raster shading and color scale creation were performed in GRASS-GIS 8.3 (https://grass.osgeo.org), map composition in QGIS 3.28 (https://qgis.org) and final artwork in Inkscape 1.3 (https://inkscape.org).

**Figure 3:**
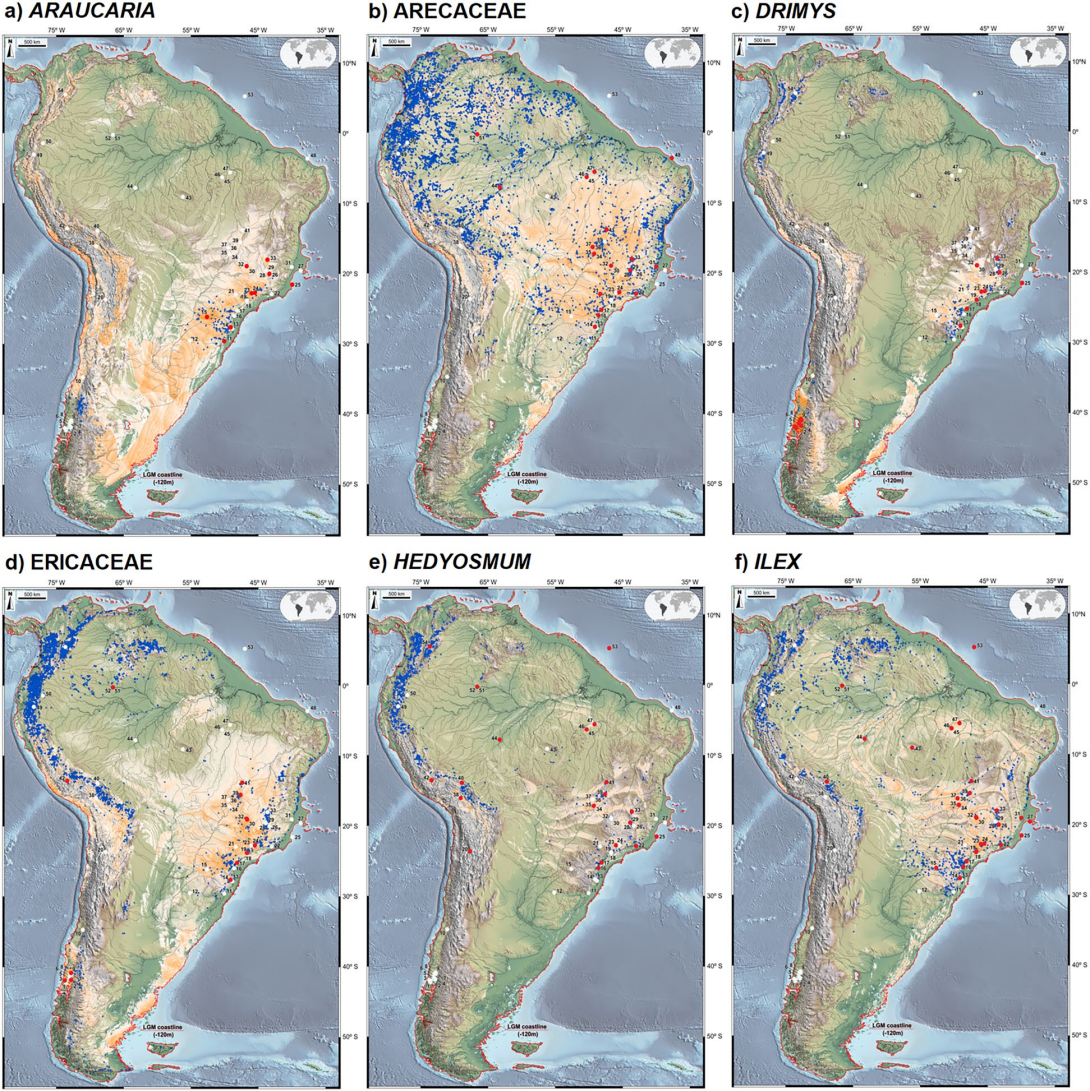
Composite maps of occurrence (numbered red dots) and absence (numbered white dots) of *Araucaria* (**a**), Arecaceae (**b**), *Drimys* (**c**), Ericaceae (**d**), *Hedyosmum* (**e**) and *Ilex* (**f**) in LGM pollen records, LGM species distribution modelling (orange to white *i.e.* high to low suitability) and modern occurrences of each taxon (blue dots). The ancient shoreline is delimited by a black line, based on sea level ca. 120 m lower than present. The base maps are shaded relief images of the ETOPO1 Global Digital Elevation Model with 01-min spatial resolution, with custom hypsometric color scales. Raster shading and color scale creation were performed in GRASS-GIS 8.3 (https://grass.osgeo.org), map composition in QGIS 3.28 (https://qgis.org) and final artwork in Inkscape 1.3 (https://inkscape.org).

**Figure 4:**
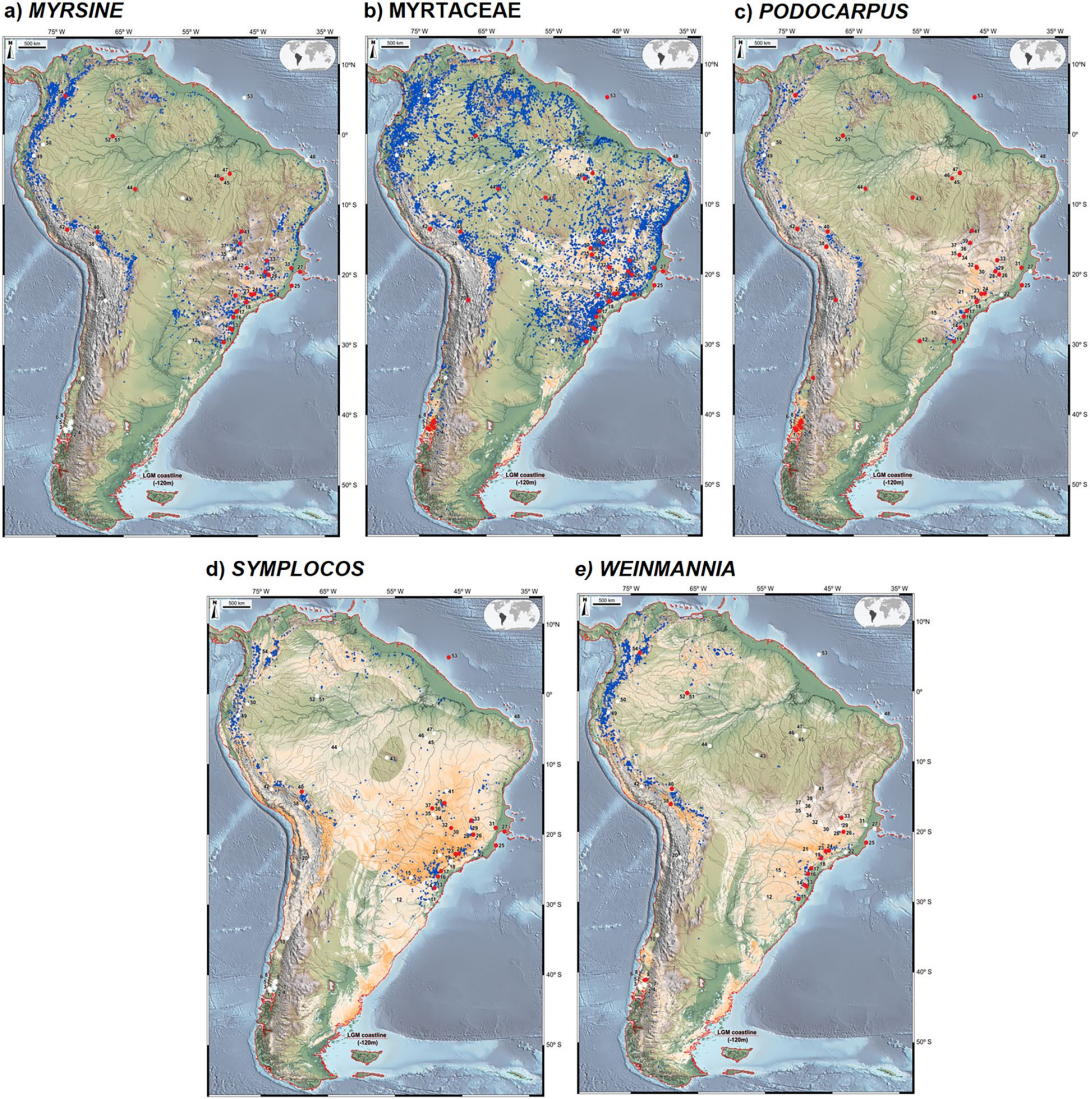
Composite maps of occurrence (numbered red dots) and absence (numbered white dots) of *Myrsine* (**a**), Myrtaceae (**b**), *Podocarpus* (**c**), *Symplocos* (**d**) and *Weinmannia* (**e**) in LGM pollen records, LGM species distribution modelling (orange to white *i.e.* high to low suitability) and modern occurrences of each taxon (blue dots). The ancient shoreline is delimited by a black line, based on sea level ca. 120 m lower than present. The base maps are shaded relief images of the ETOPO1 Global Digital Elevation Model with 01-min spatial resolution, with custom hypsometric color scales. Raster shading and color scale creation were performed in GRASS-GIS 8.3 (https://grass.osgeo.org), map composition in QGIS 3.28 (https://qgis.org) and final artwork in Inkscape 1.3 (https://inkscape.org).

Species Distribution Modelling (SDM) was carried out for each pollen taxon by means of the Maximum Entropy Method^87–89^ using MaxEnt version 3.4.4^90^, a statistical tool for the approximation of the fundamental niche of each plant taxon in terms of environmental suitability (Figs. 3, 4). SDM applied an averaging of bootstrap resampling of 30 replicates (see Supplementary Information) and was based on 19 bioclimatic variables provided by the Max Planck Institute Earth System Model for paleoclimatic conditions (MPI-ESM-P) obtained from the Climatologies at High Resolution for the Earth’s Land Surface Areas (CHELSA V1.2)^91^ dataset. Due to the complexity of the data, we have inverted the sequences of figures in order to facilitate reading, therefore the combined SDM results are synthesized in Fig. 2, depicting five connectivity patterns for the vegetation of South America during the LGM.

Present-day geographical occurrences of selected fossil pollen taxa were obtained from Global Biodiversity Information Facility (GBIF)^92^, using data cleaning^89^ procedures to check the quality of information regarding geographical coordinates followed by validation of taxonomic identification^29^. Georeferencing errors were individually evaluated and discarded. These results are given in Figs. 3 and 4 (blue dots).

**Figure 5:**
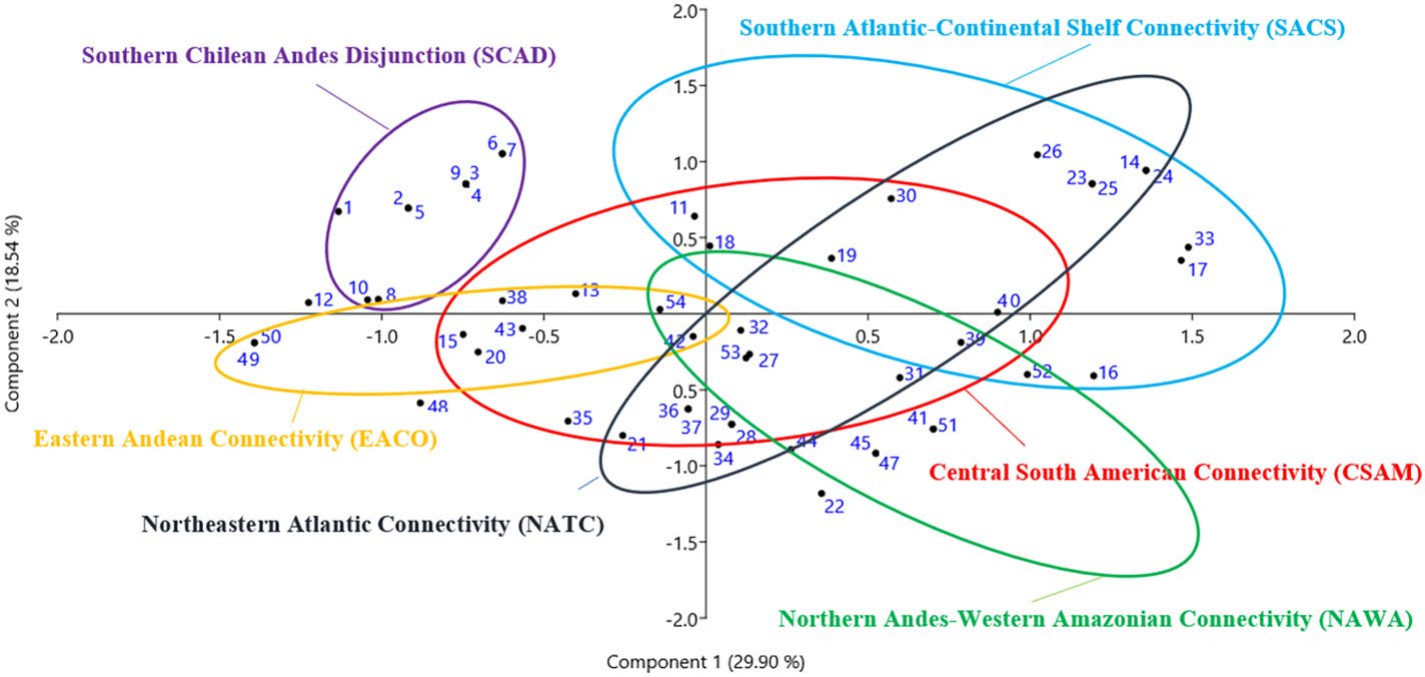
PCA clusters derived from the occurrence of selected pollen taxa in 50 LGM continental pollen records and four marine sequences. Five connectivity patterns are proposed: Central South American (CSAM), Southern Atlantic—Continental Shelf (SACS), Northern Andes—Western Amazonia (NAWA), Eastern Andean (EACO) and Northeastern Atlantic (NATC). The cluster containing pollen records 1–10, here named Southern Chilean Andes Disjunction (SCAD) is interpreted as a biogeographical region isolated from other Andean communities.

Similarity of pollen spectra in the fossil records containing the selected taxa was examined by Principal Component Analysis (PCA)^93^ via PAST 3.21^94^ software. The first two principal components (PCs) accounted for 25.95% and 18.22%, respectively, of the total variation in the dataset, resulting in a two-dimensional scatterplot of the 50 continental and 4 marine pollen record sites with data from the LGM (Fig. 5).

**Figure 6:**
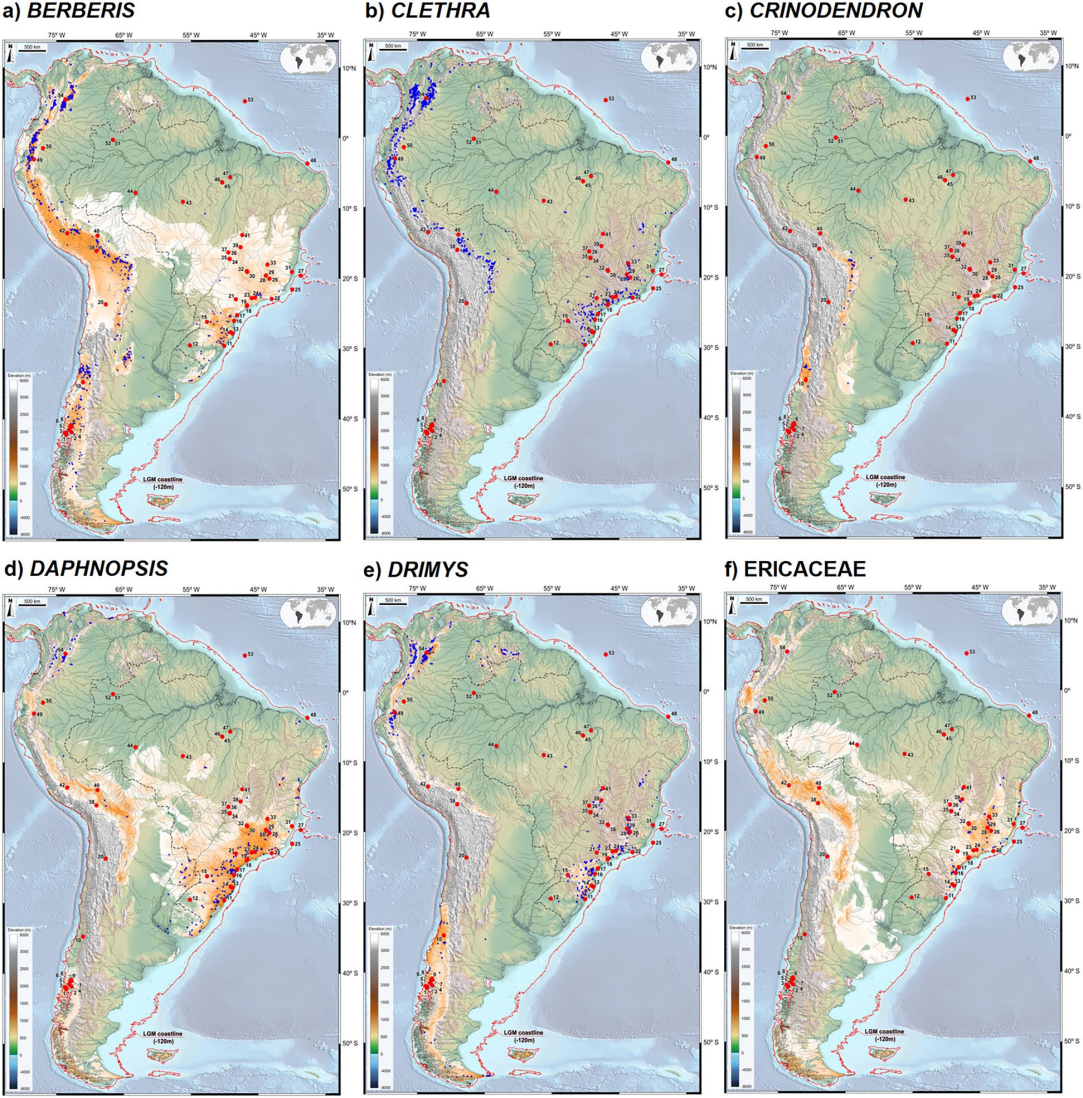
Individual maps displaying modern potential distribution for *Berberis* (**a**)*, Clethra* (**b**)*, Crinodendron* (**c**)*, Daphnopsis* (**d**), *Drimys* (**e**) and Ericaceae (**f**). Also shown are modern occurrences of each genus (blue dots), pollen record locations (numbered red dots) and the LGM coastline (red line), based on sea level ca. 120 m lower than present. The base maps are shaded relief images of the ETOPO1 Global Digital Elevation Model with 01-min spatial resolution, with custom hypsometric color scales. Raster shading and color scale creation were performed in GRASS-GIS 8.3 (https://grass.osgeo.org), map composition in QGIS 3.28 (https://qgis.org) and final artwork in Inkscape 1.3 (https://inkscape.org).

**Figure 7:**
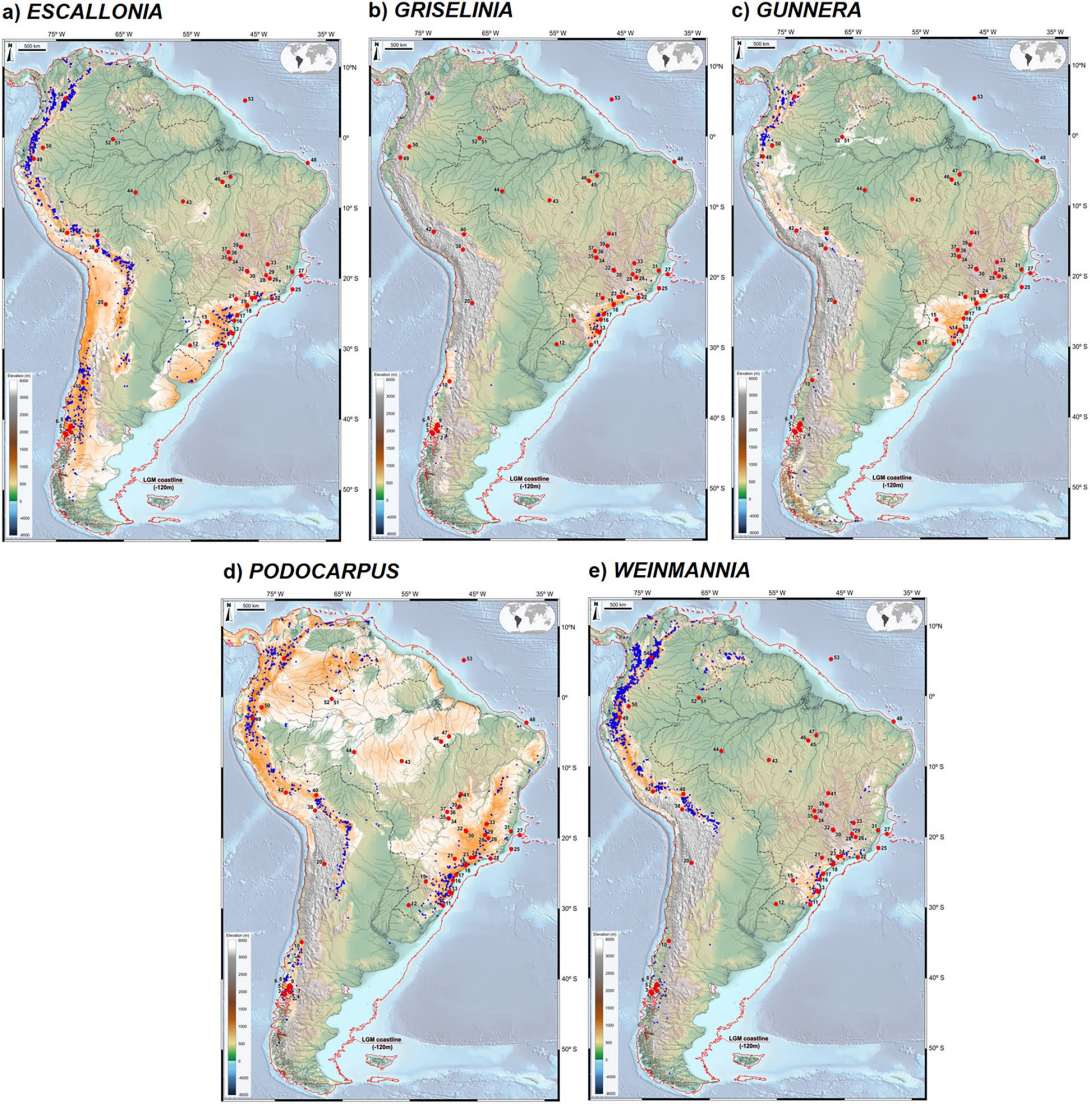
Individual maps displaying modern potential distribution for *Escallonia* (**a**)*, Griselinia* (**b**)*, Gunnera* (**c**)*, Podocarpus* (**d**) and *Weinmannia* (**e**). Each map was constructed based on the sum of potential distributions of species within each genus (orange to white, *i.e.* high to low suitability). Also shown are modern occurrences of each genus (blue dots), pollen record locations (numbered red dots) and the LGM coastline (red line), based on sea level ca. 120 m lower than present. The base maps are shaded relief images of the ETOPO1 Global Digital Elevation Model with 01-min spatial resolution, with custom hypsometric color scales. Raster shading and color scale creation were performed in GRASS-GIS 8.3 (https://grass.osgeo.org), map composition in QGIS 3.28 (https://qgis.org) and final artwork in Inkscape 1.3 (https://inkscape.org),

**Figure 8:**
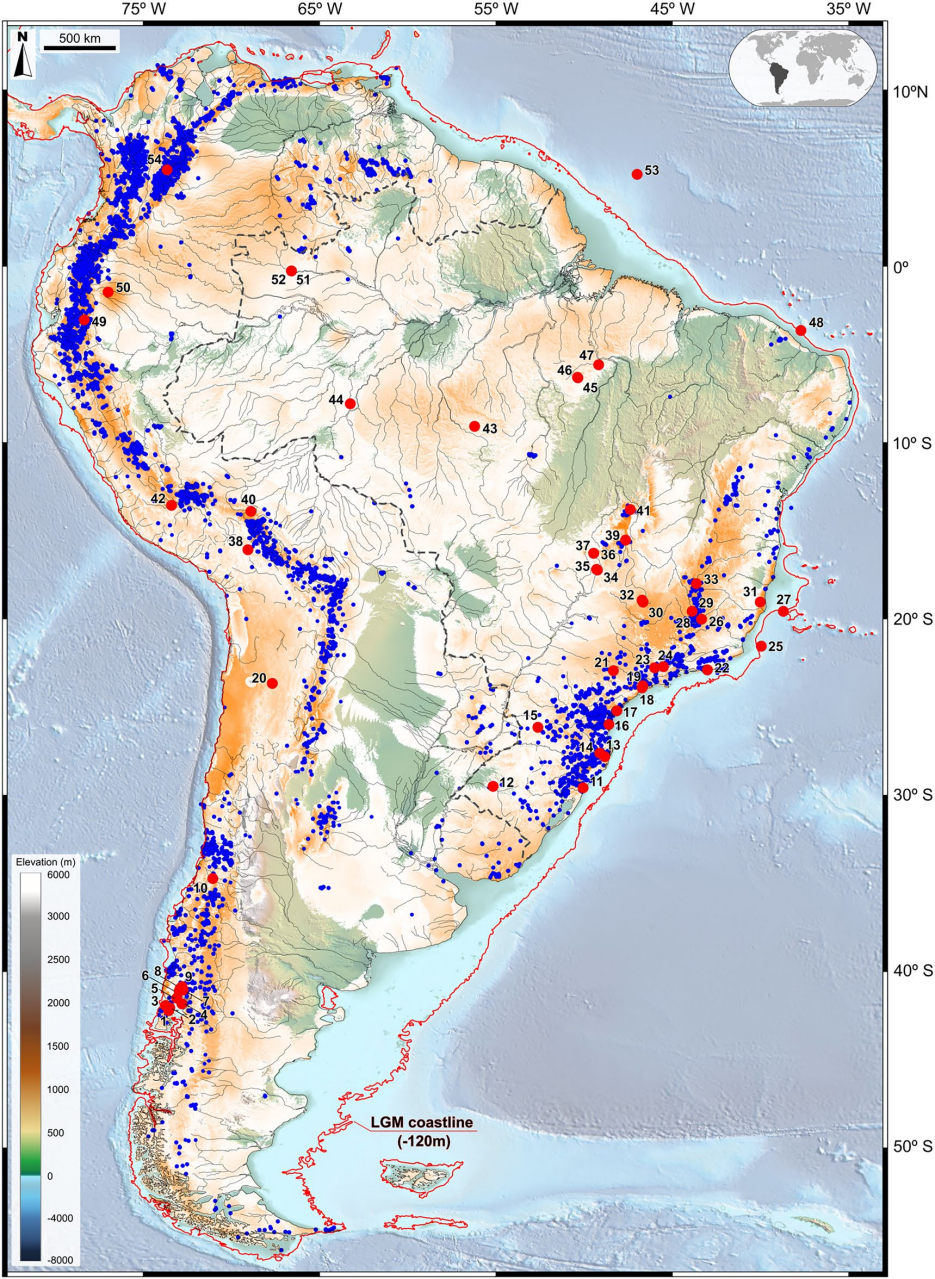
Assembled potential modern distribution of 137 selected species belonging to *Berberis, Clethra, Crinodendron, Daphnopsis, Drimys, Escallonia, Griselinia, Gunnera, Podocarpus, Weinmannia* and Ericaceae in South America supporting evidence that modern relicts of Andean and Atlantic Rainforest connections are still present in present-day landscapes (orange to white *i.e*. high to low suitability), modern plant occurrences (blue dots) 54 pollen record sites (red dots). The LGM coastline is delimited by a red line, based on sea level ca. 120 m lower than present. The base maps are shaded relief images of the ETOPO1 Global Digital Elevation Model with 01-min spatial resolution, with custom hypsometric color scales. Raster shading and color scale creation were performed in GRASS-GIS 8.3 (https://grass.osgeo.org), map composition in QGIS 3.28 (https://qgis.org) and final artwork in Inkscape 1.3 (https://inkscape.org).

In order to further corroborate the overall results, we tested the hypothesis that modern landscapes in Brazil still harbor signals of relict populations indicative of former connectivity patterns by investigating the present-day geographical distributions of 137 species (see Supplementary Information) belonging to the following Andean genera known to occur in the Atlantic phytogeographical domain: *Berberis* L.*, Clethra* Gronov. Ex L., *Crinodendron* Molina*, Daphnopsis* Mart., *Drimys* J.R. Forst. & G. Forst., *Escallonia* Mutis ex L.f., *Griselinia* Foster & Foster*, Gunnera* L., *Podocarpus* L’Hér. ex Pers., *Weinmannia* L., and members of Ericaceae. We then applied MaxEnt SDM for each of these 137 species, with averaging of bootstrap resampling of 20 replicates, based on 19 modern bioclimatic variables obtained from the CHELSA dataset. Modern occurrences for each species were obtained from GBIF^92^ by applying the same steps described above, thus generating a composite map for each genus and family (Figs. 6, 7). Finally, we assembled these results in one single map (Fig. 8) to emphasize modern signals of past contact between the Andean and Atlantic phytogeographical regions.

In order to explore paleoclimatic conditions during the LGM we inferred paleoprecipitation during the LGM by using modern analogs provided by six Brazilian indicator species: *Araucaria angustifolia* (Bert.) O. Kuntze, *Drimys angustifolia* Miers., *Drimys brasiliensis* Miers., *Hedyosmum brasiliense* Mart. ex Miq., *Podocarpus sellowii* Klotzsch ex Endl., and *Podocarpus lambertii* Klotzsch ex Endler. This approach might reduce uncertainties imposed by taxonomic identifications of pollen restricted to the genus and family levels. These six species, found across a very wide latitudinal range, are indicative of various ecosystems such as the montane Atlantic forests, the central highlands and the southern lowlands of Brazil.

**Figure 9:**
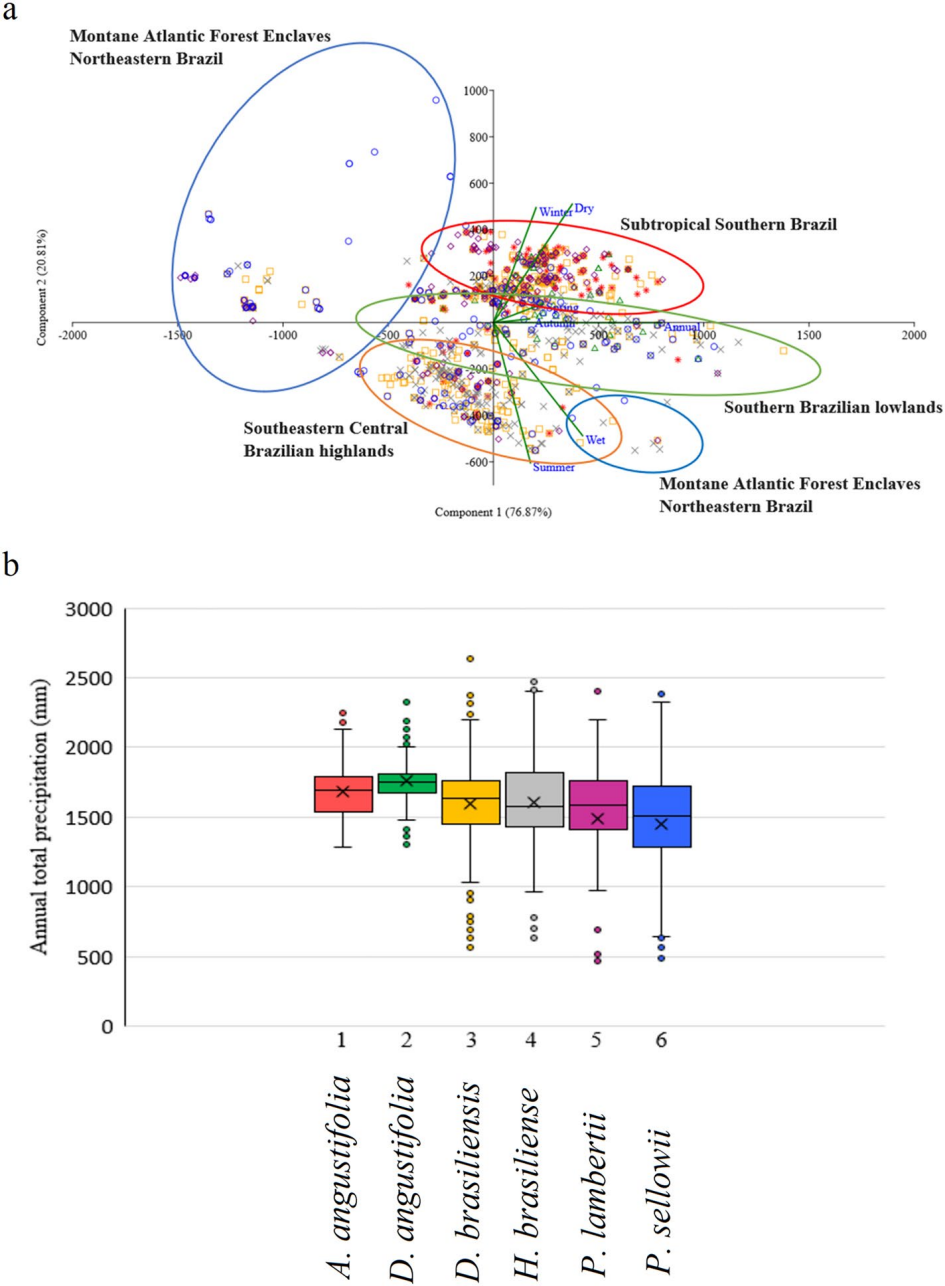
(**a**) Principal Component Analysis diagram based on the four seasons, dry and wet months and annual accumulated precipitation of each modern observed occurrence of six species: (1) *Araucaria angustifolia* (red stars), (2) *Drimys angustifolia* (green triangles), (3) *Drimys brasiliensis* (orange squares), (4) *Hedyosmum brasiliense* (gray x’s), (5) *Podocarpus lambertii* (purple diamonds), and (6) *Podocarpus sellowii* (blue circles). Four patterns in floristic composition were identified: Montane Atlantic Forest Enclaves of Northeastern Brazil (blue ellipses), Subtropical Southern Brazil (red ellipse), Southern Brazilian lowlands (green ellipse) and Southeastern Central Brazilian highlands (orange ellipse). b) Box plots show the distribution and skewness of mean annual precipitation (mm), by displaying the data quartiles and averages (‘x’ label) for each species.

Environmental distribution patterns of these six key arboreal taxa were examined by two principal components accounting for 76.64% and 20.81% of the variance, respectively, with a bidimensional scatterplot for 2231 modern occurrences (Fig. 9a). This analysis was based on four seasons (spring, summer, autumn and winter), Dry and Wet periods and annual accumulated precipitation (mm) during a 15-year period (2000–2015), for each georeferenced occurrence of each species. In addition, the box plots show the distribution and skewness of mean annual precipitation (mm), by displaying the data quartiles and averages for each species (Fig. 9b). Climate layers were provided by the Climate Prediction Center Morphing Technique (CMORPH)^95^, integrated with Brazilian Meteorological Station Network data^96–99^. We also made histograms of accumulated precipitation (mm) and calculated the mean accumulated precipitation (mm) for each species via scripts in the R^100^ language (Fig. S1, in Supplementary Information).

## Results

Integrating fossil pollen occurrences, niche and modern plant distribution data allows us to propose five LGM connections between Andean and Atlantic ecosystems and one biogeographical disjunction (Fig. 2). We here abandon the terms Cerrado and Chaco connections^101^ in favor of geographical names which do not imply floristic compositions. We hypothesize that the selected plant taxa with long-distance pollen transport and seed dispersal, such as anemophily (airborne pollination), zoochory (seed dispersal by animals), entomophily (insect pollination) and anemochory (seed dispersal by wind) syndromes^5^, had an adaptive advantage under the relatively cool and moist climates of the LGM. We envision these connections as microrefugia alignments of nuclei of cool-adapted vegetation functioning as sources of immigrants to other microrefugia along these routes, permitting geneflow between these populations, a scenario very similar to what has been proposed for northeastern Brazil during the final stages of the last glacial cycle^5^. We believe that glaciation in the high Andes did not affect our proposed connectivity patterns as these were being formed after the 1,000 m tree line descend towards the lowlands^28,33,73,74^.

### Connections revealed by pollen records

From an ecological perspective, PCA permitted a simplified vision of the spatial distribution during the LGM of the 11 selected taxa found in the 54 fossil pollen records. The resulting elliptical clusters (Fig. 5) suggest five connectivity patterns and one biogeographical disjunction. We argue that the clear overlapping of connectivity clusters is a consequence of floristic similarities between pollen diagrams at different fossil sites, driven by cool glacial climates, as follows:

*Central South American Connectivity (CSAM)* is illustrated by Arecaceae, Ericaceae, *Hedyosmum, Ilex, Myrsine*, Myrtaceae, *Podocarpus* and *Symplocos* (Figs. 3, 4)*,* where niche suitability for these taxa is highest in central Brazil. This large area lies between 13 and 30°S, containing the previously suggested Cerrado and Chaco independent connections^101^, stretching from the eastern flanks of the modern Andean Tropical Forest to its Atlantic counterparts.

Andean sites present in this cluster, within this connectivity, are Laguna Miscanti, Chile (site 20), Lake Titicaca, Bolivia (site 38), Lake Consuelo, Peru (site 40), Lake Pacucha, Peru (site 42), San Juan Bosco and Mera, Ecuador (sites 49 and 50), and Lake Fúquene, Colombia (site 54). It is noteworthy that Serra Negra (Site 32, ca. 1200 m elev.) and Turfa de Inhumas (sites 36–37, 761 m elev.) in Central Brazil are shown close to Mera, Ecuador (site 50, ca 1100 m elev.) and Pacucha, Peru (site 42, ca. 3000 m elev.), thus revealing high floristic similarities suggestive of a possible ecological corridor between these regions which was lost during the Holocene with the expansion of the modern cerrado^3,63–65,69^.

It’s important to notice that the LGM record of Serra Negra, although interpreted as a time of drier climates due the absence of a complete glacial sequence, contains relatively abundant cold-humid arboreal elements during its final stages at approximately 17 cal ka. This scenario is further supported by pollen records available for the Serra Negra region, in central Brazil, at Serra do Salitre (site 30) and Lagoa dos Olhos (sites 28, 29). These are indicative of forested landscapes with significant arboreal pollen sums, although interpreted as indicative of relatively drier climates^4,13^. In the first, Myrtaceae and Ericaceae and abundant together with aquatic vegetation dominated by Cyperaceae whereas in Lagoa dos Olhos, LGM pollen spectra are characterized by *Myrsine*, Myrtaceae, *Podocarpus* and *Araucaria*. The same humid regional trend is found at Serra do Espinhaço (site 33) with a mixture of semi-deciduous (*Cedrella*, *Tabebuia*), cold and humid forest taxa, the latter interpreted as relicts of former colder and humid climates^53^—forming non-analog assemblages with expanded Cerrado/savanna elements.

The pollen record of Pato Branco (site 15), relatively close to the southern Andes, shows high similarity with those of Lake Titicaca (site 38, *ca.* 3800 m elev.) and Lake Miscanti (site 20, *ca.* 4140 m elev.), thus supporting a wide corridor encompassing the formerly described as Cerrado and Chaco connections based on avian genetic evidence^101^.

These results imply relatively high precipitation levels during the LGM, which corroborate proposed scenarios of enhanced landslide-dammed lake formation from 40 to 25 cal ka in the northern Argentinian Andes^102^ as well as widespread humidity rendering the Altiplano of Bolivia and Peru wetter than presently^9^.

We envision these connectivities as vegetational mosaics where taxa displaying long-distance dispersal abilities were in transit and thus coupling isolated cold-humid upland forested microrefugia. This is not in contradiction with reported savanna expansion, based on pollen analyses^103,104^ nor with drier climatic scenarios based on speleothem geochemical proxies in the Pantanal macroregion^105^. In fact, the increase of savanna vegetation in certain regions might have been fundamental to the formation of some non-analog plant assemblages, especially in ecotonal domains.

The Northern Andes-Western Amazonian Connectivity (NAWA) is characterized by the occurrence of 8 study sites located at high elevations in the eastern Andes (42, Lake Pacucha, Peru, 3095 m elev.; 54, Fúquene, Colombia, 2540 m elev.) and in the Amazonian lowlands (51–52, Lake Pata, ca. 300 m elev.; 45–47, Carajás, 471 m and 119 m, respectively; 44, Humaitá, 100 m and site 53, Amazon Fan (marine)^72^. Such clustering is likely to be a consequence of pollen diagrams containing relatively high percentages of *Podocarpus* and *Hedyosmum* in association with Arecaceae, Ericaceae, *Ilex*, *Myrsine*, Myrtaceae and *Weinmannia* (Figs. 3, 4, 5). In Lake Pata, at ca. 400 m elevation and 0° latitude in western Brazilian Amazonia, these montane elements were earlier interpreted as a result of downward migration of the Pico da Neblina montane forests into the lowlands, thus resulting in non-analog plant assemblages^23,106^. A similar mechanism was also proposed much earlier as an explanation for the occurrence of Andean forest elements, such as *Alnus*, *Podocarpus*, making up new floristic associations with lowland taxa in Ecuadorian Amazonia^31,32^ involving *Alnus*, *Hedyosmum* and *Podocarpus* (site 49, San Juan Bosco and 50, Mera, ca 300 m elev. in Ecuador).

Higher percentages of *Alnus* during the LGM in marine sediments of site 53 (Amazon River Delta) are likely to represent long-distance pollen signatures exported by the Amazon Basin during high precipitation events prevailing during the LGM^9,23,31,32^.

Although present in the previous connectivity pattern, the Humaitá site (site 44, ca 100 m elev.) in southwestern Brazilian Amazonia shows a large increase in *Alnus* pollen reaching ca. 10% during the LGM. This threshold, although considered a background effect in Colombian Andean lakes^79^, can hypothetically be interpreted as active migration of this arboreal taxon into lowlands of the Brazilian Amazon. Outlier cluster points such as sites 27 and 31 in southeastern coastal Brazil and sites 32 and 39, in the Cerrado region of central Brazil, are again signaling floristic similarities brought about by cooling and higher humidity levels, which characterize most of the South American lowlands during the LGM. On the other hand, sites 49 and 50 (San Juan Bosco and Mera, in Ecuador) are outside this cluster by an artifact caused only by the presence of Ericaceae pollen, indicative of cool and moist environments.

The relatively close proximity of the marine record (Jaguaribe River Delta—GeoB 3104-1, site 48) to Amazonian sites (Humaitá, site 44 and Serra dos Carajás, site 46–47) can possibly be explained by close floristic similarities imposed by the expansion of cold- and humid-adapted arboreal taxa during the LGM as well as by long-distance pollen transport to coastal marine deposits.

Such connectivity between the Andes and the Amazon could have only been possible if one assumes a large LGM rainforest cover within most of the Amazon Basin, dominated by cold- and humid-adapted taxa, a scenario that has recently been proposed based on a biome modelling analysis for paleovegetation cover^107^^.^ All pollen spectra related to this connectivity are interpreted as a consequence of downward migration of high-elevation taxa into the Amazonian lowlands during the LGM, forming non-analog plant assemblages whose key taxa are now found with disjunct distributions in the Neblina region of the Upper Rio Negro, in Brazil^17^.

Southern Atlantic Continental Shelf Connectivity (SACS) is best represented by *Araucaria* and *Drimys* (Fig. 3), forming plant assemblages with Arecaceae, Ericaceae, *Ilex*, *Myrsine*, Myrtaceae, *Podocarpus*, *Symplocos* and *Weinmannia*. It includes Atlantic Rainforest sites extending from the Mantiqueira Mountains, the modern cerrado region of Minas Gerais into the Serra do Mar highlands along the coast of southeastern and southern Brazil: Cambará do Sul (site 11, ca. 1 m elev.), Serra do Tabuleiro (site 13, 849 m elev.), Ilha do Cardoso (site 17, 8 m elev.), Volta Velha (site 16, 8 m elev.), Curucutu (site 18, 780 m elev.), and Colônia Crater (site 19, 760 m elev.). Considering the LGM landscape, this floristic connectivity extended from the Central South American Connection (CSAM) onto the then-exposed Atlantic Shelf spanning from 23 to 56°S in latitude. We hypothesize that this large exposed area was vegetated by cold and humid successional forests as a consequence of the downward migration of montane taxa into the coastal lowlands, a scenario supported by pollen records (sites 17 and 30) from modern mangrove areas in southeastern Brazil^52,60^. A similar mechanism of rainforest and mangrove vegetation belts expanding and contracting in synchrony with sea level oscillations has been observed for the mid Holocene of coastal northeastern Brazil^108^.

SACS is characterized by a denser niche suitability in the southern Brazilian and Argentinian-Uruguayan sections of the Atlantic Shelf. Despite the apparent connection in terms of habitat suitability, the absence of *Araucaria* in the LGM pollen records of Chile might represent a significant retraction of *Araucaria araucana* (Mol.) K. Koch, the endemic Chilean species, in synchrony with a hypothetical expansion of *Araucaria angustifolia* into Uruguay and northern Argentina, especially onto the exposed continental shelf. *Drimys*, on the other hand, appears to display high niche suitability centered in southern/southeastern Brazil, on the Argentinian shelf and in a large area between 36 and 48°S on the Pacific coast of Chile. *Drimys winteri* JR Forst. & G. Forst., an Andean endemic, could have populated the latter two regions whereas areas of high habitat suitability in Brazil could have been exploited by *D*. *brasiliensis* and *D*. *angustifolia*, vicariant counterparts.

Another possible branch of this connection is suggested by higher habitat suitability during the LGM displayed by *Araucaria*, Ericaceae, *Symplocos*, *Weinmannia* and Arecaceae in northern Argentina, Paraguay and Uruguay, south of the Chaco region. The absence of pollen records south of site 11 (Cambará do Sul) limits our evaluation of this region as a potentially important area of contact between the Andes and the Atlantic.

Eastern Andean Connectivity (EACO), best represented by Ericaceae, *Hedyosmum*, *Myrsine*, *Podocarpus* and *Symplocos* (Figs. 3–5), encompasses the Ecuadorian sites of San Juan Bosco and Mera (sites 49, 50)**,** the Peruvian site of Lake Consuelo** (**site 42), Lake Titicaca (site 38), and Laguna Miscanti (site 20) in northern Chile. Noteworthy is the presence, within this cluster, of the Brazilian site of Pato Branco (15), relatively close to the Andes and the borderline site Sao Francisco de Assis (12) in coastal Brazil, at 91 m elevation which can be explained by forest spectra dominated by *Podocarpus*. The absence of Ericaceae and *Symplocos* in most of these Andean sites can be hypothesized to be a consequence of paramo vegetation expansion downslope during the LGM, generating pollen spectra dominated by grasses and few arboreal elements^68,69^.

Northeastern Atlantic Connectivity (NATC), typified by Arecaceae, Ericaceae, *Hedyosmum*, *Ilex*, *Myrsine*, Myrtaceae, *Podocarpus Symplocos* and *Weinmannia*, includes records of a trident-shaped corridor rooted at the Mantiqueira/Serra do Mar mountainous chain in Southeastern Brazil (Fig. 2), having a right-handed extension onto coastal northeastern Atlantic Forest, with its central arm reaching the modern caatinga areas and its far-left extension connecting it with southeastern Amazonia. This LGM scenario closely resembles the cold migration corridors for multiple montane forest refugia proposed for the Heinrich Stage 1 climatic phase, ca. 14–18.7 cal ka of southeastern Brazil^5^, which in turn suggests that these migration events may have also occurred during other glacial cycles.

Southern Chilean Andes Disjunction (SCAD) includes pollen records from the Pacific side of the Chilean Andes (sites 1 to 10), at elevations ca. 100–400 m, such as Lake Tagua-Tagua (site 10) at 195 m elev. The disjunct pattern is likely to represent a relatively isolated forest ecosystem created by the Andean geographical barrier containing *Drimys*, Ericaceae, Myrtaceae, *Podocarpus* and *Weinmannia*. The modern landscape of this area, between sites 9 and 10, is characterized by the southern Chile endemic *Araucaria araucana* as the dominant taxa in the emergent forest canopy, a species which surprisingly is not present in the LGM pollen records. A tentative explanation relies on the absence of palynological studies covering this glacial phase in these higher southern latitudes. It may also be that this taxon migrated to ice-free areas without lake basins or peatbogs to capture a local pollen history.

### Analysis of modern distributions as influenced by former climates: Are relicts still there?

We approached the question of whether relict populations can still be found in present-day landscapes by investigating the modern distributions of 137 species belonging to Andean genera occurring in the Atlantic domain and evaluated their ecological niches by means of SDM (MaxEnt). Each map (Figs. 6, 7) was constructed based on the sum of potential distributions of species within each genus (orange to white, *i.e.* high to low suitability).

The results indicate that modern SDM of *Berberis boliviana*, *B. ciliaris*, *B. conferta*, *B. laurina* e *B. densifolia* control most of the linkage between the two forest domains depicted on Fig. 6a, despite their absence in plant databases of central Brazil. The same applies to *Clethra brasiliensis*, *C. scabra* (*Clethra* Fig. 6b); *D. brasiliensis*, *D. coriacea*, *D. sellowiana*, *D. utilis* (*Daphnopsis*, Fig. 6d); *Gaylussacia densa*, *G. reticulata* (Ericaceae, Fig. 6f); *Escallonia angustifolia*, *E. laevis*, *E. megapotamica* (*Escallonia*, Fig. 7a); *Griselinia ruscifolia* (Griselinia, Fig. 7b); Gunnera manicata (*Gunnera*, Fig. 7c); *Podocarpus aracensis*, *P. brasiliensis*, *P. celatus*, *P. salicifolius*, *P. sellowii*, *P. transiens* (*Podocarpus*, Fig. 7d); *Weinmannia glabra*, *W. haenkeana*, *W. humilis*, *W. karsteniana*, (*Weinmannia*, Fig. 7e).

There are two possible hypothesess that could explain the absence of these species in database. The first could represented biased caused by field sampling and taxonomical identification methodologies. The second, and very plausible alternative explanation invokes habitat loss caused by unsuitable climatic change after the LGM.

Table 1 depicts the results of data integration validating LGM connections across South America with the presence of modern relicts and strengthened by a composite map of their geographical distributions (Fig. 8). *Podocarpus* and *Weinmmania*, and to a lesser degree *Escallonia* and *Berberis*, appear to be the main drivers in delimiting relicts which can be explained by their long-distance dispersal of pollen and seeds. *Podocarpus*, in particular, shows a wide environmental amplitude, occurring across a broad latitudinal and longitudinal range in South America, from 10°N to 50°S and 35°W to 80°W.

**Table 1:** Taxa occurrences in modern relicts and LGM fossil pollen records in relation to proposed connectivity patterns between the Andean and Atlantic domains.

Connectivity pattern	Modern relicts	LGM Fossil Record
CSAM	*Berberis*, *Daphnopsis*, Ericaceae, *Escallonia*, *Podocarpus*	Ericaceae, *Podocarpus*, *Weinmannia*
EACO	*Berberis*, *Clethra*, *Crinodendron*, *Daphnopsis*, *Drimys*, Ericaceae, *Escallonia*, *Gunnera, Podocarpus*, *Weinmannia*	*Drimys*, Ericaceae, *Podocarpus*, *Weinmannia*
NATC	*Clethra*, *Daphnopsis*, *Drimys*, Ericaceae, *Podocarpus*, *Weinmannia*	*Drimys*, Ericaceae, *Podocarpus*, *Weinmannia*
NAWA	*Clethra*, *Daphnopsis*, *Drimys*, Ericaceae, *Podocarpus*, *Weinmannia*	*Drimys*, Ericaceae, *Podocarpus*, *Weinmannia*
SACS	*Berberis*, *Clethra*, *Crinodendron*, *Daphnopsis*, *Drimys*, Ericaceae, *Escallonia*, *Griselinia, Gunnera, Podocarpus*, *Weinmannia*	*Drimys*, Ericaceae, *Podocarpus*, *Weinmannia*
SCAD	*Berberis*, *Crinodendron*, *Drimys*, Ericaceae, *Escallonia*, *Griselinia, Gunnera, Podocarpus*, *Weinmannia*	*Drimys*, *Podocarpus*, *Weinmannia*

Number of species used in modeling: *Berberis* (33), *Clethra* (8), *Crinodendron* (2), *Daphnopsis* (7), *Drimys* (5), Ericaceae (3), *Escallonia* (26), *Griselinia* (2), *Gunnera* (6), *Podocarpus* (20), *Weinmannia* (25).

### Paleoclimatic conditions during the LGM by using modern analogs

PCA of the modern distributions of *Araucaria angustifolia*, *Drimys angustifolia*, *D. brasiliensis*, *Hedyosmum brasiliense*, *Podocarpus lambertii* and *P. sellowii* in Brazil in relation to present-day precipitation regimes reveals five clusters associated with four connectivity patterns during the LGM (Fig. 9), as follows:

The Southeastern Central Brazilian highlands (orange ellipse) (19°–25°S) cluster is associated with the Central South American Connectivity (CSAM) within the Atlantic Forest domain, with prevalence in wet and coastal regions populated by *A. angustifolia*, *D. angustifolia*, *D. brasiliensis*, *Hedyosmum brasiliense*, *P. lambertii* and *P. sellowii*.

The Subtropical Southern Brazil (20°–25°S, red ellipse) and Southern Brazilian lowlands (25°–35°S, green ellipse) clusters are associated with the Southern Atlantic-Continental Shelf Connectivity (SACS) in southeastern Brazilian highlands and lowlands, respectively, with prevalence of *Araucaria angustifolia* (red stars), *D. brasiliensis* (orange squares), *D. angustifolia* (green triangles), *Hedyosmum brasiliense, P. lambertii* (purple diamonds) and *P. sellowii* (blue circles).

In addition, PCA revealed two clusters of montane Atlantic Forest enclaves in northeastern Brazil (4°–20°S, blue ellipses), separated mostly by annual accumulated precipitation and prevalence of wide latitudinal distributions of *Podocarpus sellowii* (blue circles) and associated with the Northeastern Atlantic Connectivity (NATC).

The statistical analysis of annual accumulated precipitation in observed occurrences identified differences in tolerance ranges for each species: *A. angustifolia*, ca. 1700 mm; *D. angustifolia*, ca. 1750 mm; *D. brasiliensis*, ca. 1600 mm; *H. brasiliense*, ca. 1600 mm; *P. lambertii*, ca. 1500 mm, and *P. sellowii*, ca. 1450 mm. Annual accumulated precipitation histograms for each species are presented in Fig. 9b and Supplementary Information Fig. S1.

These results allow us to suggest that paleoprecipitation within the SACS connectivity during the LGM in terms of mean annual accumulated precipitation was on the order of ca. 1700 mm. Because CSAM and NATC connections lacked *Araucaria* in their domains, we estimate their mean annual precipitation to be ca. 1500 mm.

### Exposed South American Atlantic continental shelf during the LGM

An important fact in South American paleovegetational studies is the impact on the ecology of coastal forests by the global sea level reduction of ca. 120–150 m^34–38^ and the emergence of the South American continental shelf. We estimated a total exposed area of 1.94 million km^2^, equivalent in size to the combined areas of France, Spain, Germany, Italy and the United Kingdom and that in southeastern/southern Brazil/Uruguay and Argentina the past coastline was 200–250 km and 500 km distant from its present location, respectively.

This notable addition to the continental ecosystem, equivalent to the area of a large portion of western Europe, could have possibly played a role in the migration process of cool and montane elements under an ecological scenario of niche expansion. A somewhat similar successional mechanism has been observed in the Atlantic rainforest region of northeastern Brazil during sea level fluctuations of the Mid-Holocene with expansions of rainforest and mangrove elements in synchrony with sea level oscillations^108^.

This ample niche opening for the Atlantic Rainforest, especially to cold and humid-adapted taxa, possibly favored *Araucaria* and *Drimys* in the higher southern latitudes of the shelf. An approximation of the possible architecture of this forest is available for lower latitudes via palynological information from the states of Sao Paulo and Espirito Santo.

Niche suitability analysis, represented by Potential distribution Modelling, during the LGM, of pollen records of Brejo do Louro^60,61^ (site 30) and Ilha do Cardoso^51,52^ (site 17), in areas presently at sea level and covered by mangrove vegetation, suggests the presence of a vertically stratified subtropical to temperate forest with distinct non-analogous, under cold and humid conditions. The data suggest a forest physiognomy composed of 5 tree layers, based on the average height of the tallest taxon within each genera or family: an emergent layer of 45–50 m composed very likely of *Araucaria angustifolia* and *Virola*, a novel combination of taxa, above a 30-m main canopy of *Alchornea*, *Cordia*, *Didymopanax* and Urticaceae/Moraceae, over a 20-m subcanopy of *Croton*, *Eriotheca*, *Euplassa*, *Ilex*, Melastomataceae, *Myrsine*, Myrtaceae, *Podocarpus*, *Protium*, *Sloanea* and *Sapium*, followed by a 10-m understory composed of small trees and shrubs of *Drimys*, Ericaceae, *Hedyosmum*, *Symplocos*, *Tapirira* and *Weinmannia*. The forest floor was likely covered by *Cyathea* tree ferns, Polypodiaceae ground ferns, and other herbs.

We argue that such forest was likely the result of the ability of cold-adapted taxa to migrate downward from the southeastern Brazilian highlands, covering considerable distances on the exposed continental shelf. Such migration capacity could have been enhanced by advantageous anemophilous and zoophilous pollen and zoochorous seed dispersal syndromes^5^ during periods of favorable climatic change with cooling and sufficient precipitation during the LGM. We believe that the combined forces of these syndromes appear to have been the motor that drove the emergence of novel plant communities with no modern analogs and that other morphological characters and physiological aparatusses must have played an important role in their ability to survive in the new connectivities’ territories. A summary of reproductive strategies for each individual pollen taxa is presented on Table 2.

**Table 2:** Plant reproductive strategies in each connectivity pattern.

Connectivity	Name	Pollen taxa, pollination and dispersal syndromes
CSAM	Central South American Connectivity	Arecaceae^a,b^, Myrtaceae^b^, *Ilex*^b^, *Hedyosmum*^a^*, Podocarpus*^a^*, Ericaceae*^b^*, Weinmannia*^c^
SACS	Southern Atlantic Continental Shelf Connectivity	*Araucaria*^a^, *Drimys*^b^, Arecaceae^a,b^, Ericaceae^b^, *Ilex*^b^*, Podocarpus*^a^*, Symplocos*^b^*, Weinmannia*^c^*,* Myrtaceae^b^
EACO	Eastern Andean Connectivity	Ericaceae^b^*, Hedyosmyum*^a^*, Podocarpus*^a^*, Myrsine Weinmannia*^c^
NAWA	Northern Andes-Western Amazonian Connectivity	Arecaceae ^a,b^, *Hedyosmum*^a^*, Ilex*^b^*, Podocarpus*^a^, Ericaceae^b^*,*Myrtaceae^b^, *Symplocos*^b^ *Weinmannia*^c^
NATC	Northeastern Atlantic Connectivity	Arecaceae^a,b^*,* Myrtaceae^b^*, Hedyosmum*^a^*, Ilex*^b^*, Podocarpus*^a^*, Symplocos*^b^*, Weinmmania*^c^
SCAD	Southern Chilean Andes Disjunction	*Drimys*^b^, *Podocarpus*^a^*,* Ericaceae^b^, Myrtaceae^b^, *Weinmannia*^c^

Pollen and seed dispersal syndromes^5^: ^a^Anemophily, zoochory, ^b^Entomophily, zoochory, ^c^Entomophily, anemochory.

This same pattern of forest formation can be applied to the exposed Amazonian portion of the South American continental shelf based on LGM pollen spectra present in offshore marine sediments (site 53) indicating the presence of a cool and moist tropical forest. Its floristic composition was dominated by the montane taxa *Podocarpus*, *Weinmannia*, *Myrica*, *Symplocos*, *Hedyosmum* and *Alnus*, also suggestive of a downslope migration into the lowlands. A recent analysis of pollen signatures transported by Amazonian rivers to its delta^109^ suggests nearby terrigenous sources as the main component in marine sediments, which implies that cold and humid forests were thriving in fluvial valleys of eastern Amazonia and by consequence on the exposed coastal shelf during the LGM. A similar scenario of large scale regional vegetation coverage was reported for the LGM of the exposed Southeast Asia-Australasia continental shelf with the predominance of rainforest in wetter areas, resulting in a greater area of rainforest than nowadays^110^.

### Non-analogous plant assemblages

The combined LGM MaxEnt niche suitability maps for all fossil taxa indicate that the proposed connectivity patterns were characterized by expanded ranges of cold‐tolerant forest taxa, leading to the establishment of a series of plant assemblages without modern analogs. In the SACS and CSAM connections, for instance, cold-adapted montane taxa appear more prevalent at the expense of Cerrado elements as depicted in the Lagoa Bonita (site 39), Chapada dos Veadeiros (site 41), Lagoa dos Olhos (sites 28 and 29), and Serra do Salitre (site 30) records. During the LGM these taxa, currently restricted to cool and humid montane ecosystems, thrived sympatrically along with Cerrado elements such as *Antonia ovata* Pohl, *Byrsonima* L., *Caryocar* A. St.-Hill, *Emmotum* Benth., *Kielmeyera* (Spr.) Mart., *Mauritia* L., *Neea* Ruiz & Pav., *Ouratea* Aublet, *Qualea* Mart., *Stryphnodendron* Mart. and *Vochysia* Aublet, among others. Another significant example is given by *Araucaria* and *Podocarpus* thriving sympatrically during the LGM with Cerrado elements at Lagoa Santa in south Central Brazil^18^ and during most of the last glacial cycle (ca. 90–23 cal ka) of the Serra Negra record^3^. This wide scenario of multiple migrations of subtropical taxa during the final stages of the last glacial cycle offers a plausible explanation for the modern disjunct occurrences of *Podocarpus* in the semi-deciduous tropical rainforest region of São Paulo^111–113^ and in the semi-arid caatinga domain of Bahia^114,115^.

It is worth mentioning that SDM and modern occurrences especially of *Podocarpus* and *Hedyosmum* in the tepui region of southern Venezuela and northern Brazilian Amazonia (sites 51–52, Lagoa da Pata) depict possible former connections between these areas under a scenario of 1100 m downslope migration^23,116^ of cold-adapted taxa, forming plant assemblages of warm- and cold-adapted taxa living sympatrically^23,106,117^ and without modern counterparts.

In summary, we envision these multiple phases of mixed temperate plant assemblages of glacial age in South America as a parallel to the well-established phases of generalized migration of temperate forest taxa in North America^118,119^ adjusting to the rearrangement of their fundamental niches driven by climatic changes related to Marine Isotope Stage 2 (MIS 2; ~ 29–14 ka).

### Former climatic mechanisms and northward displacement of cold air masses

Our overall data confirm that during the Last Glacial Maximum, cold fronts were intensified and could have reached farther north than at present, thus affecting northeastern Brazil. Because the resulting equator-pole temperature gradient was therefore larger, transient systems had probably higher intensity thus causing convergence of humidity precisely where the mean humidity zonal stream flow was high^13,15,120,121^.

In this period, in the regions near the equator, the changes in humidity and temperature were smaller and, at high latitudes, larger, with lower temperatures and less humidity. Circulations such as Rossby waves were likely to be intensified and amplified, which resulted in the propagation of cold fronts and associated cold air masses to the equatorial region^13,97,98,122^. This scenario is corroborated by recent cases of the incursion of cold waves from the high latitudes of the southern hemisphere that even cross the equator because of the break in the geostrophic balance caused by the Andes^123–128^. The pressure gradient is maintained (high temperature contrast) with decreasing Coriolis force (decreasing wind)^13^, thus enhancing the cold air mass acceleration northwards. These cold outbreak events, denominated in Brazil as “friagens”, could have been intensified during glacial periods of the South American continent.

### Genetic and additional palynological evidence for a late Quaternary Andean-Atlantic connection

A wide array of evidence for historical connections between Andean and Atlantic ecosystems has been provided by genetic analyses in certain groups of animals, such as birds^101,129–133^, frogs^134^ and rodents^135^. Among these studies, there is significant support for a major disjunction between the Andes and the Atlantic Forest between 1 Ma and 0.15 Ma, with multiple cycles of connectivity through the Chaco-Southern Brazil and Cerrado, in Central Brazil^101,136,137^. Other analyses also based on genetic flow between closely related bird species with Andean-Atlantic disjunct distributions focused on the LGM as a more recent decisive moment of connectivity, under humid phases and expansion of forests in both the Cerrado and Chaco routes^129^.

Genetic analyses of two bird species (*Syndactyla rufosuperciliata* and *S. dimidiata*) occurring in both regions suggest genetic flow through two main migration corridors between the Andes and coastal Brazil^129^: one connecting the southern Andes to the Atlantic via southern Brazil, known as the Chaco Connection, along the main rivers in that region, *i.e.*, the Bermejo and Pilcomayo Rivers. A second route occurred in central Brazil. The authors propose the hypothesis that the connection between the Andean and the Atlantic regions occurred through past forest expansions (*i.e.*, gallery and or semideciduous) in the Cerrado region, and call this the Cerrado Connection^101,137^. Additional analysis of past distributions of *Podocarpus*, *Ilex*, *Hedyosmum* and *Myrsine*^138^, plant genera also occurring in the Restinga vegetation, a sub-set ecosystem of the Atlantic Rainforest, has shown that Central Brazil functioned as a node of migration between the Amazon and Atlantic rainforests, linking the Andes to the central and coastal mountains of Brazil, during glacial periods.

## Conclusions

Our study provides strong evidence for the establishment of ecological corridors linking Andean, Atlantic and Amazonian regions under the relatively cool and moist climates of the LGM, which favored the migration of various plant and animal groups. The largest of these connections, spanning from 10 to 30°S in latitude, corroborates previous biogeographic and genetic studies in animal taxa proposing a Chaco-Southern Brazil and Cerrado corridor connected to Andean ecosystems. North of this domain, a Northern Andes-Amazonian connection was characterized by forest elements that probably migrated downwards into the lowlands, benefiting from cooler and humid climates and forming non-analogous forest assemblages. This mechanism can therefore provide an explanation for increased presence of *Alnus* pollen in a glacial pollen record in the Amazonian lowlands. Evidence of this expansion is also found in marine pollen records of the LGM. The significant sea level fall of ca. 120 m exposed the South American Continental Shelf, which could have created an important migration corridor for different southern Andean plant species to migrate northwards and colonize areas of the Brazilian Atlantic coast. Our data suggests that this vast coastal corridor was possibly covered by temperate-like forest with prevalent Andean floristic affinities in the south and a more Atlantic floristic composition in the north. This study also brings to light a discussion on plant ecological niches with the establishment of novel plant assemblages with non-modern analogs, which appears to be a common feature of glacial vegetations under cold and moist climates.

### Supplementary Information

Supplementary Information.

## Data Availability

All data generated or analysed during this study are included in this published article (and its Supplementary Information files).
