# Supplementary material for: Humid and cold forest connections in South America between the eastern Andes and the southern Atlantic coast during the LGM
Source: Sci Rep. 2024 Jan 24;14:2080. doi: 10.1038/s41598-024-51763-8 (PMC10808232; doi:10.1038/s41598-024-51763-8)
Supplement: Supplementary file 1 — Supplementary Information. [file 41598_2024_51763_MOESM1_ESM.docx]

**Supplementary Information**

**Humid and cold forest connections in South America between the eastern Andes and the southern Atlantic coast during the LGM**

Pinaya, Jorge Luiz Diaz^1,2*^; Pitman, Nigel C.A.^3^, Cruz, Francisco William^1^; Akabane, Thomas K. ^1^; Lopez, Maria del Carmen Sanz^4^; Pereira-Filho, Augusto José^4^; Grohman, Carlos H.^5^; Reis, Luiza Santos^1^; Rodrigues, Erika S. Ferreira^1^; Ceccantini, Gregório C.T.^6^ and De Oliveira, Paulo Eduardo^1,3*^

^1^Institute of Geosciences, University of São Paulo, Brazil.

^2^Polytechnic School, University of São Paulo, Brazil.

^3^Science Action, The Field Museum of Natural History, Chicago, Illinois, USA.

^4^Instituto de Astronomia, Geofísica e Ciências Atmosféricas, Universidade de São Paulo, Brazil.

^5^Institute of Energy and Environment, University of São Paulo, Brazil.

^6^Institute of Biosciences, Dept. of Botany, University of São Paulo, Brazil.

*email: jorge.pinaya.usp@gmail.br; paulo.deoliveira@usp.br

**SI1 Complementary information of Figure 2-4: List of 11 family and genus in species distribution modeling under LGM climate conditions**

The potential distribution maps for each family and genus under LGM climatic conditions resulted from species distribution modeling of 11 family and genus in South America. The name of taxa and the summaries of results from 30 bootstrap models for each taxon under LGM climate conditions, using Maxent version 3.4.1, are presented following. We present the average training AUC for the replicated runs and the standard deviation.

*Araucaria* (AUC = 0.945, Std = 0.026), *Drimys* (AUC= 0.987, Std=0.006), *Hedyosmum* (AUC= 0.973, Std=0.015), *Ilex* (AUC=0.983, Std=0.008), *Myrsine* (AUC=0.981, Std=0.006), *Podocarpus* (AUC= 0.974, Std=0.012), *Symplocos* (AUC=0.946, Std=0.023), *Weinmannia* (AUC=0.955, Std=0.014), Myrtaceae (AUC=0.981, Std=0.008), Ericaceae (AUC=0.967, Std=0.017), Arecaceae (AUC=0.940, Std=0.030).

**SI2 Complementary information of Figure 6-7: List of 137 species used in species distribution modeling under modern climate conditions**

The potential distribution maps for each family and genus under modern climatic conditions resulted from compositing the individual species distribution modeling of 137 species in South America, using Maxent version 3.4.4. The name of specie and the summaries of results from 20 bootstrap models for each taxon under LGM climate conditions, using Maxent version 3.4.1, are presented following. We present the average training AUC for the replicated runs and the standard deviation.

*Berberis* (33 species): *Berberis actinacantha* (AUC=0.996, Std=0.001), *Berberis agapatensis* (AUC=0.990, Std=0.004), *Berberis boliviana* (AUC=0.937, Std=0.028), *Berberis bumeliifolia* (AUC=0.995, Std=0.003), *Berberis carinata* (AUC=0.997, Std=0.002), *Berberis chilensis* (AUC=0.998, Std=0.000), *Berberis ciliaris* (AUC=0.996, Std=0.002), *Berberis commutata* (AUC=0.983, Std=0.006), *Berberis conferta* (AUC=0.947, Std=0.042), *Berberis darwinii* (AUC=0.994, Std=0.002), *Berberis densa* (AUC=0.991, Std=0.006), *Berberis densifolia* (AUC=0.993, Std=0.004), *Berberis dumaniana* (AUC=0.974, Std=0.019), *Berberis empetrifolia* (AUC=0.992, Std=0.001), *Berberis ferruginea* (AUC=0.996, Std=0.004), *Berberis glauca* (AUC=0.990, Std=0.004), *Berberis goudotti* (AUC=0.994, Std=0.001), *Berberis grandiflora* (AUC=0.991, Std=0.004), *Berberis hallii* (AUC=0.996, Std=0.001), *Berberis hieronymi* (AUC=0.997, Std=0.001), *Berberis huertasii* (AUC=0.991, Std=0.005), *Berberis jamesonii* (AUC=0.998, Std=0.0)*, Berberis laurina* (AUC=0.985, Std=0.003), *Berberis lehmannii* (AUC=0.988, Std=0.010), *Berberis lutea* (AUC=0.992, Std=0.001), *Berberis quindiuensis* (AUC=0.995, Std=0.004), *Berberis rariflora* (AUC=0.993, Std=0.004), *Berberis rigida* (AUC=0.998, Std=0.001), *Berberis rigidifolia* (AUC=0.997, Std=0.002), *Berberis ruscifolia* (AUC=0.996, Std=0.001), *Berberis Saxicola* (AUC=0.988, Std=0.0), *Berberis tabiensis* (AUC=0.993, Std=0.004), *Berberis verticillate* (AUC=0.996, Std=0.001);

*Clethra* (8 species): *Clethra brasiliensis* (AUC=0.979, Std=0.014), *Clethra cardenasii* (AUC=0.995, Std=0.002), *Clethra castaneifolia* (AUC=0.994, Std=0.002), *Clethra cuneata* (AUC=0.991, Std=0.002), *Clethra fagifolia* (AUC=0.980, Std=0.001), *Clethra fimbriata* (AUC=0.985, Std=0.002), *Clethra revoluta* (AUC=0.980, Std=0.001), *Clethra scabra* (AUC=0.968, Std=0.002);

*Crinodendron* (2 species): *Crinodendron tucumanum* (AUC=0.999, Std=0.0), *Crinodendron patagua* (AUC=0.992, Std=0.003);

Daphnopsis (7 species): *Daphnopsis brasiliensis* (AUC=0.982, Std=0.009), *Daphnopsis caracasana* (AUC=0.899, Std=0.115), *Daphnopsis coriacea* (AUC=0.968, Std=0.017), *Daphnopsis fasciculata* (AUC=0.988, Std=0.005), *Daphnopsis racemosa* (AUC=0.982, Std=0.003), *Daphnopsis sellowiana* (AUC=0.983, Std=0.014), *Daphnopsis utilis* (AUC=0.994, Std=0.002);

*Drimys* (5 species): *Drimys brasiliensis* (AUC=0.988, Std=0.002), *Drimys granadensis* (AUC=0.916, Std=0.149), *Drimys roraimensis* (AUC=0.983, Std=0.029), *Drimys winteri* (AUC=0.954, Std=0.020), *Drimys angustifolia* (AUC=0.996, Std=0.002);

Ericaceae (3 species): *Gaylussacia densa* (AUC=0.967, Std=0.018), *Gaylussacia reticulata* (AUC=0.996, Std=0.002), *Gaylussacia brasiliensis* (AUC=0.994, Std=0.002);

*Escallonia* (26 species): *Escallonia alpina* (AUC=0.990, Std=0.002), *Escallonia angustifolia* (AUC=0.954, Std=0.033), *Escallonia bifida* (AUC=0.988, Std=0.002), *Escallonia cordobensis* (AUC=0.997, Std=0.001), *Escallonia discolor* (AUC=0.991, Std=0.007), *Escallonia farinacea* (AUC=0.997, Std=0.000), *Escallonia hypoglauca* (AUC=0.994, Std=0.002), *Escallonia illinita* (AUC=0.998, Std=0.000), *Escallonia laevis* (AUC=0.987, Std=0.006), *Escallonia leucantha* (AUC=0.997, Std=0.001), *Escallonia megapotamica* (AUC=0.981, Std=0.006), *Escallonia millegrana* (AUC=0.991, Std=0.004), *Escallonia mutis* (AUC=0.972, Std=0.006), *Escallonia myrtilloides* (AUC=0.967, Std=0.002), *Escallonia myrtoidea* (AUC=0.997, Std=0.001), *Escallonia paniculata* (AUC=0.972, Std=0.001), *Escallonia pendula* (AUC=0.989, Std=0.001), *Escallonia pulverulenta* (AUC=0.987, Std=0.007), *Escallonia resinosa* (AUC=0.986, Std=0.005), *Escallonia reticulata* (AUC=0.998, Std=0.000), *Escallonia revoluta* (AUC=0.989, Std=0.009), *Escallonia rosea* (AUC=0.995, Std=0.001), *Escallonia rubra* (AUC=0.989, Std=0.002), *Escallonia schreiteri* (AUC=0.982, Std=0.014), *Escallonia tucumanensis* (AUC=0.987, Std=0.009), *Escallonia virgata* (AUC=0.991, Std=0.002);

*Grisellinia* (2 species): *Griselinia scandens* (AUC=0.989, Std=0.002), *Griselinia ruscifolia* (AUC=0.998, Std=0.001);

*Gunnera* (6 species): *Gunnera bogotona* (AUC=0.971, Std=0.033), *Gunnera brephogea* (AUC=0.836, Std=0.136), *Gunnera magellanica* (AUC=0.904, Std=0.088), *Gunnera manicata (AUC=0.964, Std=0.035), Gunnera Pilosa* (AUC=0.991, Std=0.021), *Gunnera atropurpurea* (AUC=0.918, Std=0.101);

*Podocarpus* (20 species): *Podocarpus aracensis* (AUC=0.940, Std=0.028), *Podocarpus brasiliensis* (AUC=0.979, Std=0.007), *Podocarpus celatus* (AUC=0.971, Std=0.043), *Podocarpus coriaceus* (AUC=0.994, Std=0.017), *Podocarpus glomeratus* (AUC=0.988, Std=0.015), *Podocarpus guatemalensis* (AUC=0.992, Std=0.005),  *Podocarpus lambertii* (AUC=0.993, Std=0.001), *Podocarpus magnifolius* (AUC=0.964, Std=0.016), *Podocarpus nubigenus* (AUC=0.998, Std=0.001), *Podocarpus oleifolius* (AUC=0.951, Std=0.115), *Podocarpus parlatorei* (AUC=0.928, Std=0.088), *Podocarpus roraimae* (AUC=0.968, Std=0.031), *Podocarpus rusbyi* (AUC=0.993, Std=0.004), *Podocarpus salicifolius* (AUC=0.953, Std=0.027), *Podocarpus salignus* (AUC=0.999, Std=0.001), *Podocarpus sellowii* (AUC=0.977, Std=0.005), *Podocarpus sprucei* (AUC=0.999, Std=0.001), *Podocarpus steyermarkii* (AUC=0.978, Std=0.020), *Podocarpus tepuiensis* (AUC=0.993, Std=0.004), *Podocarpus transiens* (AUC=0.978, Std=0.016);

*Weinmannia* (25 species): *Weinmannia auriculata* (AUC=0.991, Std=0.004), *Weinmannia auriculifera* (AUC=0.986, Std=0.007), *Weinmannia balbisiana* (AUC=0.982, Std=0.001), *Weinmannia brachystachya* (AUC=0.997, Std=0.000), *Weinmannia crassifolia* (AUC=0.991, Std=0.002), *Weinmannia cundinamarcensis* (AUC=0.993, Std=0.003), *Weinmannia elliptica* (AUC=0.989, Std=0.002), *Weinmannia engleriana* (AUC=0.996, Std=0.003), *Weinmannia fagaroides* (AUC=0.979, Std=0.002), *Weinmannia glabra* (AUC=0.978, Std=0.006), *Weinmannia guyanensis* (AUC=0.989, Std=0.008), *Weinmannia haenkeana* (AUC=0.982, Std=0.010), *Weinmannia hirtella* (AUC=0.989, Std=0.005), *Weinmannia humilis* (AUC=0.981, Std=0.010), *Weinmannia karsteniana* (AUC=0.973, Std=0.019), *Weinmannia latifolia* (AUC=0.989, Std=0.004), *Weinmannia laurina* (AUC=0.982, Std=0.009), *Weinmannia laxiramea* (AUC=0.996, Std=0.002), *Weinmannia lechleriana* (AUC=0.987, Std=0.004), *Weinmannia lentiscifolia* (AUC=0.985, Std=0.007), *Weinmannia loxensis* (AUC=0.998, Std=0.000), *Weinmannia macrophylla* (AUC=0.996, Std=0.000), *Weinmannia mariquitae* (AUC=0.990, Std=0.001), *Weinmannia microphylla* (AUC=0.990, Std=0.003), *Weinmannia multijuga* (AUC=0.984, Std=0.003).

*.*

(1) (2)


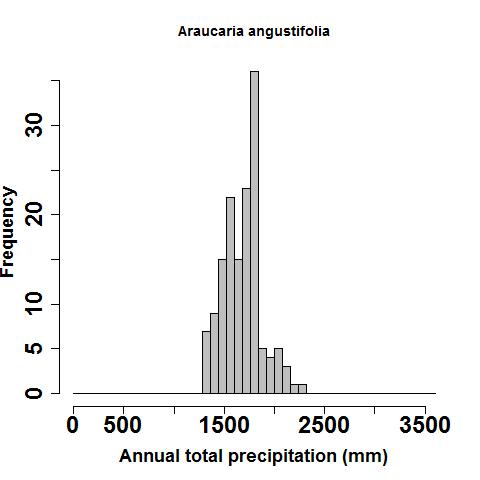

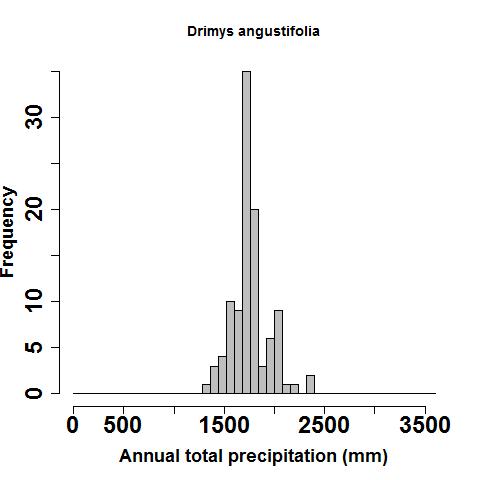


(3) (4)


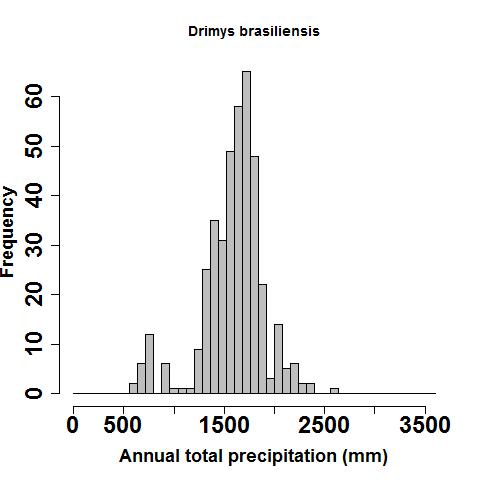

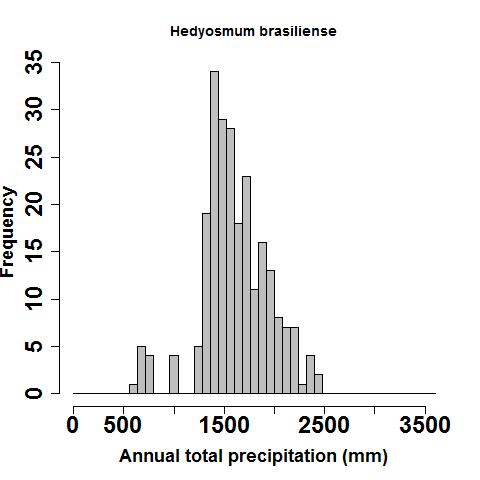


(5) (6)


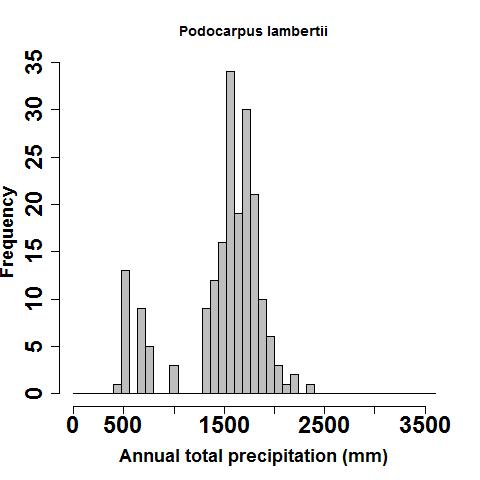

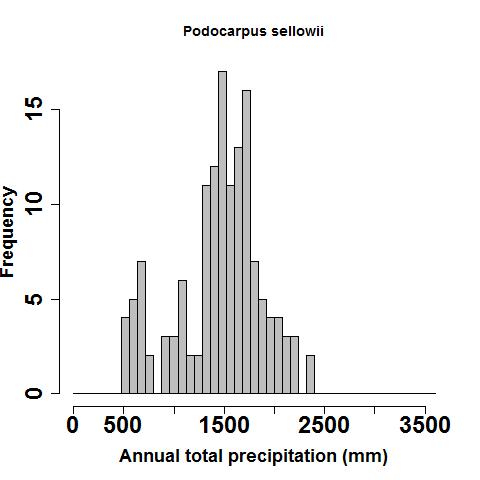


Figure S1: Histograms of annual accumulated precipitation of modern observed occurrences of six South American tree species: (1) *Araucaria angustifolia*, (2) *Drimys angustifolia*, (3) *Drimys brasiliensis*, (4) *Hedyosmum brasiliense*, (5) *Podocarpus lambertii*, and (6) *Podocarpus sellowii*.
